# Extraction and characterisation of arabinoxylan from brewers spent grain and investigation of microbiome modulation potential

**DOI:** 10.1007/s00394-021-02570-8

**Published:** 2021-05-31

**Authors:** Kieran M. Lynch, Conall R. Strain, Crystal Johnson, Dhrati Patangia, Catherine Stanton, Fatma Koc, Jorge Gil-Martinez, Patrick O’Riordan, Aylin W. Sahin, R. Paul Ross, Elke K. Arendt

**Affiliations:** 1 School of Food and Nutritional Sciences, University College Cork, Cork, Ireland; 2 Teagasc Food Research Centre, Fermoy, Co., Cork, Ireland; 3 APC Microbiome Ireland, University College Cork, Cork, Ireland; 4 Global Innovation and Technology Centre, Anheuser-Busch InBev nv/sa, Brouwerijplein 1, 3000 Leuven, Belgium

**Keywords:** Arabinoxylan, Fibre, Microbiome, Prebiotic

## Abstract

**Purpose:**

Brewers’ spent grain (BSG) represents the largest by-product of the brewing industry. Its utilisation as an animal feed has become less practical today; however, its high fibre and protein content make it a promising untapped resource for human nutrition. BSG contains mainly insoluble fibre. This fibre, along with protein, is trapped with the complex lignocellulosic cell structure and must be solubilised to release components which may be beneficial to health through modulation of the gut microbiota.

**Methods:**

In this study, the application of a simultaneous saccharification and fermentation process for the extraction and solubilisation of arabinoxylan from BSG is demonstrated.

**Results:**

Processing of the BSG was varied to modulate the physicochemical and molecular characteristic of the released arabinoxylan. The maximum level of arabinoxylan solubilisation achieved was approximately 21%, compared to the unprocessed BSG which contained no soluble arabinoxylan (AX). Concentration of the solubilised material produced a sample containing 99% soluble AX. Samples were investigated for their microbiome modulating capacity in *in-vitro* faecal fermentation trials. Many samples promoted increased *Lactobacillus* levels (approx. twofold). One sample that contained the highest level of soluble AX was shown to be bifidogenic, increasing the levels of this genus approx. 3.5-fold as well as acetate (*p* = 0.018) and propionate (*p* < 0.001) production.

**Conclusion:**

The findings indicate that AX extracted from BSG has prebiotic potential. The demonstration that BSG is a source of functional fibre is a promising step towards the application of this brewing side-stream as a functional food ingredient for human nutrition.

**Supplementary Information:**

The online version contains supplementary material available at 10.1007/s00394-021-02570-8.

## Introduction

As a society, the global population faces a number of challenges related to food systems and the consumption today; these include a situation where consumers often chose highly refined or processed food products, of which up to 16% is needlessly discarded [1]. Alongside this, from public health and nutrition perspectives, the world is facing a ‘fibre gap’, with approx. 80% of consumers not ingesting the recommended daily amount of fibre of 25 g/day [2–4]. Confounding these issues, by 2050, there will be an estimated global population of nearly 10 billion, yet suitable arable land on which to grow crops to feed the world becomes scarce [5]. Therefore, as consumers and as manufacturers, there should be emphasis on reducing food loss and food wastage.

Brewers spent grain (BSG), the largest by-product of the brewing industry, represents a raw material whose re-imagined utilisation and valorisation could address, at least in part, some of the issues outlined above. It accounts for 85% of the total by-product arising from brewing operations, with approx. 39 million tonnes produced globally per annum [6]. The material is the spent, fibrous outer grain layers of the barley kernels that remain after the extraction of starch and other carbohydrates during the brewing process. Thus, BSG is low in mono- and disaccharides which are solubilised and lost into the wort and high in fibre; on a dry-matter basis BSG is approx. 30–50% non-starch polysaccharides (fibre), 19–30% protein, 12–28% lignin, 10% lipids and 2–5% ash [6]. Traditionally, BSG was utilised as a feed for farm animals; however, its utilisation in this way today is becoming less practical. Many large breweries produce amounts which far outweigh the feeding requirements of the farms within the distance which it is practically, economically and safely possible to transport the BSG [7]. As a consequence of the high moisture content, it is often not practical to transport the material over long distances, coupled with the propensity of the material to spoil relatively rapidly if not processed in some way to reduce the microbial load.

In this context, investigations into ways in which this material can be valorised and (re)utilised are timely and of increasing research interest. BSG represents a valuable source of fibre. Alongside cellulose, arabinoxylan (AX) is an abundant fibre in BSG. Arabinoxylan and arabinoxylan-oligosaccharides (AXOS) from other cereal sources, primarily wheat, have been shown to be linked to potential health benefits, such as modulation of glycaemic index and potential prebiotic capacity [8–11]. Both EFSA and the FDA allow claims on AX in relation to modulating the glycaemic response; EFSA restricts this claim to wheat AX while the FDA does not [12, 13]. Despite the significant body of research surrounding wheat AX(OS), fewer studies have examined AX(OS) from barley [14–16]. One factor contributing to this, and particularly in the context of BSG, may be the recalcitrant nature of the material [17]. Whereas most of the water-extractable and soluble material will have been released and lost into the wort during the brewing process, substances remaining in the BSG are those that are primarily water-unextractable or insoluble. In addition, the complex, lignocellulosic structure of the outer grain material that represents the BSG means that the valuable AX fibre is essentially locked in a complex matrix of cellulose, AX, protein and lignin. Thus, BSG and its constituents are primarily insoluble and water-unextractable (and biologically unavailable) and must therefore be processed in some way to be released. Several processes have been investigated with this aim, and can be either physical, chemical or enzymatic processes, or combinations of these [18, 19].

The aim of this study was to examine the microbiota-modulating capacity of AX(OS) extracted from BSG. The AX extraction was performed based on a patented simultaneous saccharification and fermentation process. The time point of addition of the main xylanase enzyme (β-glucanase/pentosanase) was varied with the objective of (1) promoting AX(OS) solubility (water-extractable AX) and (2) modulating the physicochemical characteristics of the extracted AX(OS), resulting in six differently treated samples, and an untreated sample and a commercial arabinoxylan product as controls. The microbiota-modulating potential of those samples was investigated in in-vitro faecal fermentation trials after performing an in-vitro simulated digestion process followed by size exclusion chromatography to mimic intestinal absorption to remove any digestible compounds. Such a valorisation process for BSG that extracts potentially functional fibre could address a number of food system challenges: the recovery of valuable fibre for human nutrition, reducing food losses at point of manufacture and potentially positively modulating the gut microbiota which can be associated with a number of health benefits.

## Materials and methods

### Brewers spent grain production and characterisation

BSG was obtained from the AB-InBev brewery in Leuven, Belgium. The grist was composed of 60% barley and 40% rice. This spent grain was obtained from a brewing process that utilised a mash filter. Immediately after collection, the BSG was frozen at – 20 °C to preserve it until further use.

Methods for the determination of the composition of the raw material are outlined as follows: total nitrogen was quantified by Kjeldhal, A-EBC 4.3.1 [20] and using the conversion factor 6.25, the total crude protein content was determined; total fat and ash were determined according to EC 152/2009 [21]; total carbohydrate was calculated; total dietary fibre and associated components were determined according to AOAC 2011.25 [22]; sugars were determined via ion chromatography with pulsed amperometric detection; salt was calculated based on sodium (× 2.5); sodium was determined using inductively coupled plasma-optical emission spectrometry and fatty acids were determined according to ISO 5508/5509 [23]. The analyses were performed by Concept Life Sciences Ltd, UK.

### Bacterial strain and propagation conditions

The lactic acid bacteria strain, *Lactiplantibacillus plantarum* F10 (basonym: *Lactobacillus plantarum* [24, 25]) was used in this study. This strain has been isolated from malt and is retained in the culture collection of the Cereal and Beverage Science research group, University College Cork, Ireland. The strain was stored in a 40% glycerol-MRS medium at − 80 °C and routinely cultivated on de Man, Rogosa and Sharpe agar (Merck, Gillingham, UK), supplemented with 0.05 g/L bromocresol green. Incubation was performed anaerobically at 30 °C for 48 h. Single colonies were sub-cultured into MRS broth and grown overnight (15 h) as required.

### Simultaneous saccharification and fermentation of BSG

The simultaneous saccharification and fermentation (SSF) and associated thermal treatment processes were performed in 1 L bioreactor vessels of an Eppendorf DASGIP Bioblock system consisting of TC4SC4 (temperature control and agitation) and PH8 (pH monitoring) modules (Eppendorf, Julich, Germany). The samples produced and investigated are outlined in Table 1.

**Table 1:** Descriptions of samples investigated in this study

Sample #	Sample name*	Soluble AX richness (% of the total AX)	Process description
1	Untreated	N/A	BSG raw material that has not been treated in any way
2	Enzyme treated, fermented, grinded before	18.1	Sample was grinded before the SSF process Laminex addition at 70 °C during cooling from 90 °C to 35 °C
3	Enzyme treated, fermented, low WE-AX richness	11.0	No Laminex addition
4	Enzyme treated, fermented, high WE-AX richness	21.4	Laminex addition at the beginning of the SSF process at 35 °C
5	Enzyme treated, fermented, high AX richness, grinded	15.5	Sample 4, grinded after process
6	Insoluble fibre extract	N/A	High-fibre by-product from BSG
7	Soluble fibre extract	99.2	Dried powder containing soluble AX (Supernatant fraction of sample 2)
8	BioActor Naxus®	90.5	Commercial arabinoxylan (AXOS) product derived from wheat; control

*Underlined words are those used to represent the samples in subsequent figures in this paper

SSF = saccharification and fermentation process

N/A = not applicable due to water-insolubility of the sample

The SSF process applied to samples 2–5 is based on a patented process [26], with some modifications. Briefly, 200 g BSG (~ 73% moisture) was mixed with 410 mL of distilled water for a final approximate dry mater content of 10% (w/v). All samples were subjected to an initial thermal treatment of 90 °C for 30 min, followed by cooling to 35 °C at which temperature the SSF process (addition of saccharolytic enzymes and *L. plantarum* F10) was performed for 6 h.

The enzyme cocktail for saccharification consisted of 1000 ppm Laminex Super 3G (Danisco, Copenhagen, Denmark), 500 ppm Attenuzyme Pro, 100 ppm UltraFlo FABI and 200 ppm Alcalase (Novozymes, Copenhagen, Denmark). The time-point of addition of Laminex was varied in some samples as indicated in Table 1 resulting in different richness of water-extractable arabinoxylans (WE-AX). Additionally, some samples were grinded with a Silverson L4RT bench-top high shear mixer (Silverson Machines Ltd., Buckinghamshire, UK) at maximum speed for 3 min to maximise the level of solubilised AX.

For fermentation, cells of an overnight (15 h) culture of *L. plantarum* F10 were harvested by centrifugation (5000 rpm, 10 min, 4 °C), washed twice in sterile tap water (autoclaved tap water), resuspended in sterile tap water and added to each fermentation at 1% (v/v) which refers to an inoculation level of 10^7^ CFU/ml. Following the SSF process, all samples were subjected to a final pasteurisation of 70 °C for 30 min, followed by cooling and freezing at – 80 °C. Agitation was performed throughout the process at 220 rpm.

Samples 1–6 were freeze dried after production on a VirTis BenchTop Pro freeze dryer (SP Scientific, PA, USA). In addition, the supernatant fraction of sample 2 (sample 7) was freeze-dried. Naxus® is an arabinoxylan extract from wheat produced and sold by BioActor BV (Maastricht, Netherlands), and included in this study as a control. The samples were produced twice in bioreactors under controlled conditions.

### Characterisation of arabinoxylan content in samples

**Fig. 1:**
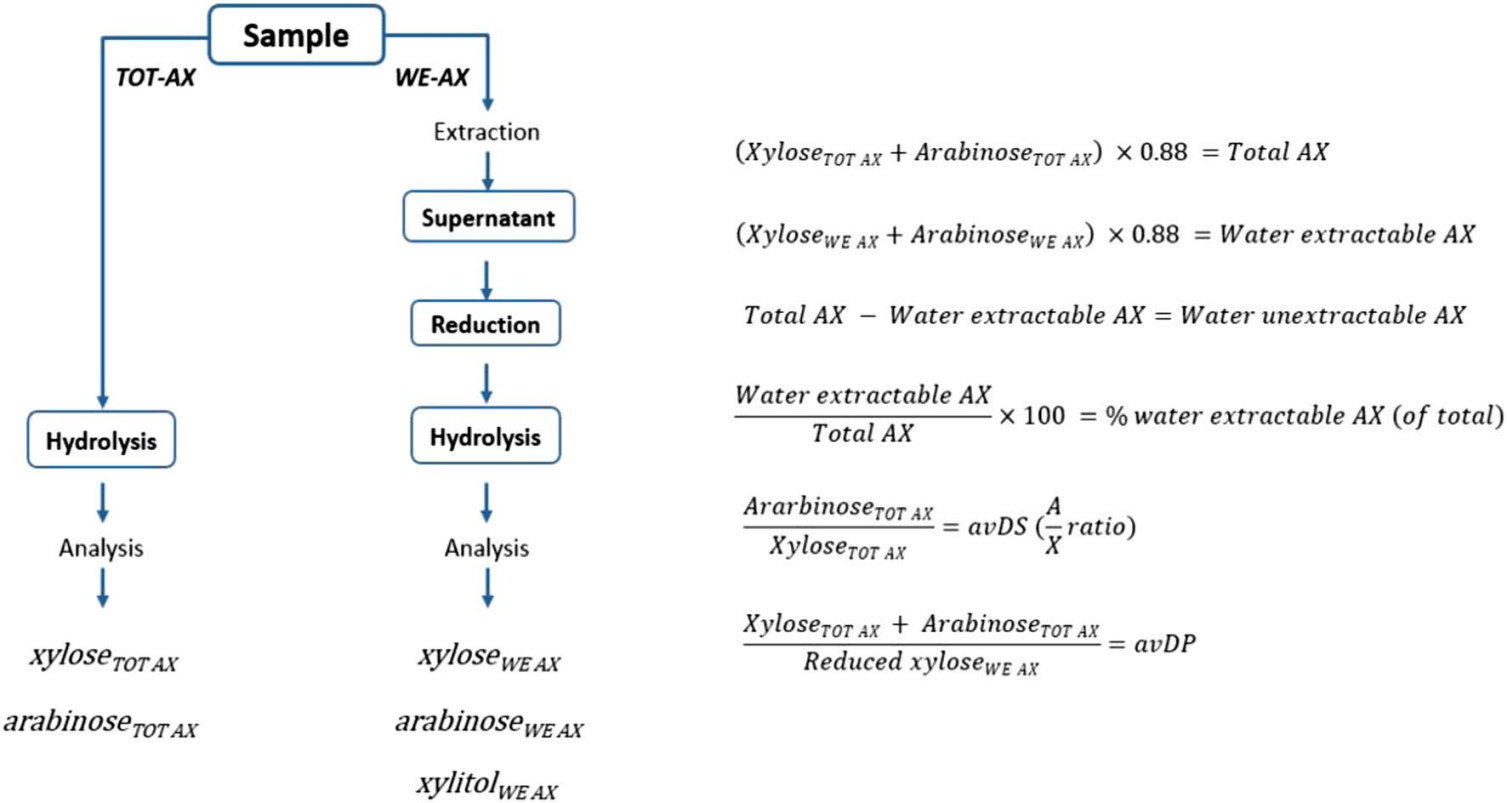
Experimental procedures and associated formulae used for characterising AX in this study

In preparation for subsequent AX analysis and characterisation, samples were frozen at – 80 °C, followed by freeze drying. Figure 1 outlines the analysis protocol applied for characterising arabinoxylans from BSG. Arabinoxylan analyses were performed in triplicate on duplicate samples.

Sample extraction was performed in milliQ water at room temperature for 30 min under agitation. For the determination of water-extractable arabinoxylan, the supernatant was retained, and a reduction was performed by mixing with ammonia (25%; Merck) and sodium borohydride (Merck). Octanol was added to prevent foaming. Samples were heated for 30 min at 40 °C, followed by the addition of acetic acid to stop the reaction. Hydrolysis of both the total sample (TOT AX) and reduced extract (WE-AX) was performed with TFA and heating to 100 °C on a block heater for 4 h. Hydrolysed samples were diluted in 20 ppm sodium azide for analysis of monosaccharides via high-performance anion-exchange chromatography coupled with pulsed amperometric detection (HPAEC-PAD). The quantification of monosaccharides was conducted via HPAEC-PAD, performed on a Dionex™ ICS-5000 + system (Sunnyvale, CA, USA) as described by Ispiryan et al. (2019) [27]. Briefly, all carbohydrates were quantified using authentic reference standards. The system was equipped with an SP Single Pump (analytical gradient pump), an ASAP Autosampler, a 10 μL of injection loop (full loop injection used), and an ED Electrochemical Detector cell with a gold working electrode and a PdH reference electrode. The eluents, purified water, 225 mM NaOH, and 500 mM NaOAc (vacuum filtered through a 0.2 μm filter), as well as the syringe wash solution, 5% CH_3_CN, were kept under a N_2_ atmosphere using a direct connection to a Peak Scientific (Inchinnan, U.K.) Corona Air Compressor and a Corona Nitrogen Generator (constant pressure of 4.5 − 5 bar). The separation was performed on a Thermo Scientific Dionex CarboPac PA1 analytical column (2 × 250 mm) with the corresponding guard column with isocratic elution with 18 mM NaOH. The compositions of the mobile phases are presented in supplementary Table 1. Separation and detection were carried out at 25 °C with a 0.25 mL/min flow rate. As results the total AX content in the individual sample, the content of water-extractable AX (WE-AX) and the amount of WE-AX of the total AX was calculated and given as the richness of WE-AX in %.

### *In-vitro* simulated gastric and intestinal digestion

An *in-vitro* fermentation system was used to explore changes in the composition and metabolic activity of human faecal microbial communities induced by the samples under investigation following simulated gastric and upper intestinal digestion. Samples listed in Table 1 were processed according to the harmonised INFOGEST protocol [28, 29] to mimic the typical phases of human digestion: oral, gastric, and intestinal digestion. Composition of Simulated Salivary Fluid (SSF), Simulated Gastric Fluid (SGF), and Simulated Intestinal Fluid (SIF) can be found in supplementary Table 2. Enzyme solutions were prepared as follows: Human salivary α-amylase solution, 1500 U mL^−1^ in SSF-ESS, Porcine pepsin stock solution, 25,000 U ml^−1^ in SGF-ESS, and Pancreatin solution, 800 U mL^−1^ in SIF-ESS.

**Table 2:** Composition of brewers’ spent grain used in this study

Parameter	Quantity (g/100 g d.m.)
Crude protein	31.0
Total fat	9.7
Ash	3.6
Total carbohydrate	12.6
Total dietary fibre	44.8
Insoluble high mol. weight dietary fibre	43.0
Total arabinoxylan	21.9
Soluble high mol. weight dietary fibre	< 1.8
Low mol. Weight dietary fibre	< 0.7
Fructose	< 0.4
Galactose	< 0.4
Glucose	< 0.4
Lactose	< 0.4
Maltose	< 0.4
Sucrose	< 0.4
Total sugars	0.4
Salt	< 0.1
Sodium	< 0.04
Energy (kcal)	350.2
Monounsaturated fatty acids	1.3
Polyunsaturated fatty acids	5.0
Saturated fatty acids	2.7
Total Trans Fatty Acids	< 0.4
Total EPA + DHA Omega-3 fatty acids	0.004
Total Omega-3 fatty acids	0.4
Total Omega-6 fatty acids	4.6
C18:3(n-3)cis Alpha-Linolenic acid (ALA)	0.4
C20:5(n-3)cis Eicosapentenoic acid (EPA)	0.004
C22:5(n-3)cis Docosapentaenoic (DPA)	< 0.002
C22:6(n-3)cis Docosahexaenoic (DHA)	< 0.002
Total Omega-9 fatty acids	1.2
Omega-3: Omega-6 Ratio	0.1

After the simulated digestion processes were completed, the end-product was dialysed (1 kDa molecular weight cut off) for 24 h to account for absorption in the small intestine. Finally, the retentate was weighed to calculate yield and freeze-dried in preparation for batch culture fermentation.

### In-vitro faecal fermentations

Stool from six healthy individuals (ages 23–50 with no antibiotic use in the 6 months prior to sample donation) was collected, pooled, homogenised, dispensed into equal parts, and frozen at – 80 °C for use in batch faecal fermentations [30]. Participant recruitment and stool samples were obtained according to ethical approval by the Clinical Research Ethics Committee of the Cork Teaching Hospitals (protocol no. APC055). Faecal samples were processed within 2 h of collection. In an anaerobic cabinet (Don Whitley anaerobic workstation), 400 g faeces were placed into large stomacher bags and homogenised with 400 mL of 50 mM anaerobic phosphate buffer. The slurry was then centrifuged at 4000 × *g* for 25 min in a Sorvall SLA-3000 rotor and the cell pellets resuspended in 400 mL of anaerobic phosphate buffer. The resulting faecal bacterial suspension was amended with sterile glycerol to a final concentration of 25% (v/v), divided into even sets of aliquots, and frozen at – 80 °C to maintain identical faecal suspensions with which to carry out all subsequent work.

### Preparation of basal media

A minimal medium adapted from Fooks and Gibson [31] was prepared as follows (mg/L): Tryptone Water, 2,000; Yeast Extract, 2,000; Cysteine HCl, 1,000; Bile Salts, 500; Tween 80, 2 mL; Hemin, 50 (dissolved in 3 drops of 1 M NaOH); Vitamin K1, 10 μL; Antifoam, 200 μL; NaCl, 100; KH_2_PO_4_, 40; K_2_HPO_4_, 40; CaCl_2_.6H_2_O, 40; MgSO_4_.7H_2_O, 10; and NaHCO_3_, 2,000. The media was autoclaved (121 °C for 15 min) and stored anaerobically until use.

### ***In-vitro*** batch fermentation

Following *in-vitro* simulated oral, gastric, and upper intestinal digestion, faecal fermentations were carried out in the MicroMatrix™ (Applikon Biotechnology, Heertjeslaan 2, 2629 JG Delft, Netherlands) 24-well cassette which enabled simultaneous individual process control, including media-only fermentation in accordance with methods provided by O’Donnell et al. [30]. Each well represented an individual bioreactor, which was seeded with 5 mL of basal fermentation medium and inoculated with 20% faecal slurry preparation (thawed for 30 min prior to use), and 0.25 g of sample under investigation in an anaerobic cabinet. Installation of the cassette and running parameters were in accordance with manufacturer’s instructions, with controlled variables including acidity (constant pH 6.8), dissolved oxygen (dO_2_ set-point 0%) and temperature (*T* = 37 °C). With six replicates carried out for each substrate. The contents of each well were mixed by means of an orbital shaker for 24 h. Fermentates were collected, centrifuged at 16,000 *g* for 10 min at 4 °C. The supernatant was collected for SCFA analysis and pellets for DNA extraction and frozen at – 80 °C.

### Short chain fatty acid analysis

Faecal fermentation effluent from each vessel was thawed on ice, syringe filtered (0.22 μm, PES, Corning) and a 270 µL aliquot was mixed with 30 µL of 10 mM of 2-ethylbutyric acid as internal standard (IS) in duplicate.

The samples were vortexed and then centrifuged at 20,000 *g* before transfer to 250 µL of glass inserts with polymer feet (Agilent, California, United States) and placed in amber glass 2 mL GC vials (Agilent, California, United States). A seven-point standard curve (acetate, propionate, butyrate and valerate (0.1 mM–10 mM); iso-butyrate and iso-valerate (0.01 mM–1 mM)) was generated from a stock solution for quantification and ran prior to the sample analysis. All standards were purchased from Sigma-Merck (Missouri, United States).

Standards and samples were analysed by gas chromatography flame ionisation detection (GC-FID) using a Varian 3800 GC system, fitted with a DB-FFAP column (30 mL × 0.32 mm ID × 0.25 μm df; Agilent, California, United States) and a flame ionisation detector. Samples and standards were loaded (0.2 µL splitless injection) with a CP-8400 autosampler (Varian, California, United States). Helium was employed as the carrier gas at a constant flow rate of 1.3 mL/min. The initial GC oven temperature was set at 50 °C, and was maintained for 0.5 min, raised to 140 °C at 10 °C/min and held for 0.5 min, before being increased to 240 °C at 20 °C/min, and held for 5.0 min (total run time 20 min). The temperatures of the detector and the injection port were set at 300 °C and 240 °C, respectively. Peaks were integrated using Varian Star Chromatography Workstation version 6.0 software.

### DNA extraction

Each pellet (from 2 mL of effluent) underwent a repeated bead beating DNA extraction according to Yu & Morrison, 2004 [32]. Chemicals were purchased from Sigma-Merck (Missouri, United States), if not stated differently.

Briefly, pellets were resuspended in 1 mL of lysis buffer (500 mM NaCl, 50 mM Tris–HCl, pH 8.0, 50 mM EDTA, and 4% sodium dodecyl sulphate) and transferred to a 2 mL of screw capped microtube (Sarstedt, Nümbrecht, Germany) containing 0.125 g of 0.1 mm and 0.125 g of 1.5 mm, a single 2.5 mm Zirconia/Silica beads (Biospec, Oklahoma, United States). The bead tubes were subjected to a bead beating step using a bead beater (Biospec, Oklahoma, United States) at 4000 rpm for 3 min. The tubes were then incubated at 70 °C for 15 min before centrifugation at 16,000 *g* for 5 min at 4 °C. The supernatant was collected and 300 µL of lysis buffer was added to the bead tube and the previous steps repeated. After pooling both supernatants, 260 µL of 7.5 M ammonium acetate solution was added, the samples were homogenised and incubated on ice for 5 min before centrifugation (16,000 *g* for 10 min at 4 °C). The supernatants were collected and mixed with equal parts of iso-propanol and stored at – 20 °C overnight. The samples were then centrifuged at 16,000 *g* for 15 min at 4 °C. The supernatant was discarded, and the pellets were washed with 200 µL of 70% ethanol, allowed to dry, before resuspending in 200 µL of Tris–EDTA buffer. The suspensions underwent two enzymatic degradation steps (RNAse 2 µL of 10 mg/mL for 15 min at 37 °C followed by Proteinase K 15 µL + 200 µL of Buffer AL (Both from the Qiagen mini stool kit) for 10 min at 70 °C. After which each sample was homogenised with 200 µL of ethanol before loading onto the DNA stool mini kit spin columns (Qiagen, Hilden, Germany) and following the manufacturer’s protocols (washed with buffers AW1 & AW2 before eluting in 200 µL of ATE buffer).

### 16S Library preparation

DNA extracts were prepared using Illumina’s 16S Metagenomic Sequencing Library Preparation guidelines. DNA samples were amplified using primers for the V3-V4 16S gene [33]. All PCR reactions were carried out using a 2720 thermal cycler (Life Technologies) with KAPA HiFi HotStart ReadyMix. The amplicon PCR program was 95 °C for 5 min, followed by 25 cycles of 95 °C for 30 s, 55 °C for 30 s and 72 °C for 30 s followed by a 5 min 72 °C elongation step before ramp down to 4 °C. The Index PCR program was the same as the amplicon step except it was carried out for 8 cycles instead of 25. All PCR products were visualised on agarose gels (1.5%, Sigma-Merck) stained with SYBR safe (Thermofisher, MA, United States). PCR reactions were cleaned using AMPure XP bead solution (Beckman Coulter, CA, USA) according to Illumina’s guidelines. Final DNA products were quantified using the Qubit dsDNA high-sensitivity kit (Thermofisher) and measured on a Qubit 3.0 fluorometer (Thermofisher).

### Bioinformatics analysis

The 300 bp paired-end FastQ reads resulting from 16S sequencing were joined using FLASH (fast length adjustment of short reads) using default parameters [34]. Demultiplexing and filtering was performed using QIIME’s split_libraries_fastq.py. USEARCH analysis tool was used for further quality filtering. Single unique reads were removed; denoising, chimera removal and grouping into operational taxonomic units (OTUs) at 97% similarity was performed using USEARCH v7 (64 bit) [35]. OTUs were aligned using Pynast (PyNAST: python nearest alignment space termination; a flexible tool for aligning sequences to a template alignment) [36]. Further, taxonomic ranks were assigned using BLAST against the SILVA SSURef database release 132 [37].

### Genome analysis of *Lactiplantibacillus plantarum* F10

The draft genome of *L. plantarum* F10 was first annotated using Prokka [38]. Furthermore, annotation was performed using dbCAN. dbCAN is a web service for automated CAZyme annotation, which integrates results from 3 databases, namely HMMER, DIAMOND and Hotpep [39]. Results from Prokka and dbCAN annotation were screened for the presence of enzymes responsible for breakdown of arabinoxylan such as L-arabinofuranosidase, ferulic acid esterase, endo-1,4-xylanase, beta-xylosidase and acetyl-xylan-esterase.

### Statistics

Statistical analysis of the AX compositional characteristics was performed as follows. Analysis was performed in R version 3.6.3. Analysis of Variance (ANOVA) was followed by pairwise comparisons by Tukey's Honestly Significant Difference (HSD) test using the agricolae package v. 1.3–2 [40]. All statistical tests were evaluated at a significance level of *p* = 0.05.

Analysis of SCFA data and alpha diversity was carried out using IBM SPSS version 24. The one-way analysis of variance (ANOVA) was carried out followed by Dunnet’s test applied for pair wise comparisons for the SCFA data and the Tukey post hoc test was employed for alpha diversity data. All other microbiome analysis and figures were carried out and generated using Calypso v8.72. The Wilcoxen rank test corrected with the Benjamini–Hochberg (BH) for multiple comparisons was employed to assess significant differences between substrates at the phylum, family and genus taxonomic levels. Principle coordinate analysis was carried out at the OUT level using the Bray Curtis metric.

## Results

### Brewers spent grain characterisation

The BSG used in this study was prepared from a grist containing 60% barely and 40% rice. Compositional analysis (Table 2) revealed the material to be high in protein (31.0 g/100 g d.m.) and dietary fibre (44.8 g/100 g d.m.), of which 43.0 g/100 g d.m. insoluble dietary fibre. The arabinoxylan concentration measured was 21.9 g/100 g d.m. As would be expected due to losses of the most soluble components in the wort, the level of total sugars (mono- and disaccharides) was very low (0.4 g/100 g d.m.). The total carbohydrate content was 12.6 g/100 g d.m. Polyunsaturated fatty acids represented the majority of analysed fatty acids (5.0 g/100 g d.m.), followed by omega-6 fatty acids (4.6 g/100 g d.m.) and saturated fatty acids (2.7 g/100 g d.m.).

### Arabinoxylan characterisation of BSG samples

Results of the arabinoxylan (AX) characterisation of the samples are shown in Table 3. The total AX concentration in the samples was 23.5% on average, except for samples 2, 7 and 8. The level in sample 2 was significantly lower. While sample 7 contained the lowest level of total AX, at 11.1%, this was found to be primarily soluble AX (99% WE-AX richness). Besides 11.1% AX, sample 7, putatively, contained low molecular weight proteins and amino acids as well as short chain polysaccharides and sugars as a result of the enzyme treatment. Naxus® (sample 8) a wheat AX extract also composed primarily of soluble AX showed the highest AX concentration (67.7 ± 3.9%) which was in correspondence with the specification provided by the supplier. Besides AX, Naxus® contains protein, ash and small amounts of lipids [41].

**Table 3:** Arabinoxylan (AX) characteristics of BSG and associated samples

Sample #	Total AX (%)	Water extractable AX (%)	WE-AX richness (% of TOT-AX)	avDP (WE-AX)	avDS (WE-AX)
1	21.9 ± 0.7^b^	N/A	N/A	N/A	N/A
2	15.8 ± 1.3^bc^	2.9 ± 0.3^b^	18.4 ± 2.0^b^	4.7 ± 0.0^c^	0.7 ± 0.0^c^
3	20.1 ± 2.3^bc^	2.2 ± 0.1^b^	11.0 ± 0.3^b^	6.3 ± 0.0^c^	1.3 ± 0.0^a^
4	25.8 ± 0.4^b^	5.5 ± 0.3^b^	21.4 ± 1.3^b^	4.8 ± 0.0^c^	0.9 ± 0.0^bc^
5	25.5 ± 1.2^b^	4.0 ± 0.1^b^	15.5 ± 0.6^b^	5.0 ± 0.0^c^	0.8 ± 0.0^c^
6	24.3 ± 1.4^b^	N/A	N/A	N/A	N/A
7	11.1 ± 0.3^c^	11.0 ± 0.2^b^	99.2 ± 2.7^a^	15.6 ± 1.0^b^	1.1 ± 0.0^ab^
8	67.7 ± 3.9^a^	61.2 ± 6.0^a^	90.5 ± 10.4^a^	33.1 ± 3.0^a^	0.8 ± 0.1^c^

The values represent the average ± standard deviation. Values in the same row with the same lower-case letter showed no significant difference (*p* < 0.05). ‘avDP’ and ‘avDS’ represent the average degree of polymerisation of water extractable arabinoxylans (WE-AX) and the average degree of arabinose substitution, respectively. Total AX refers to the total arabinoxylan content in the sample of which in % water extractable arabinoxylan are illustrated in row 3. The richness of WE-AX is related to the total arabinoxylan content of the individual sample

Differential treatment processes were performed with the objective of producing samples with different levels of soluble AX and/or AX structural characteristics, such as average degree of polymerisation (avDP) and average degree of substitution (avDS). For comparison purposes between samples, the richness of soluble AX (WE-AX yield) is a more informative parameter to discuss. Like the absolute soluble AX value, the soluble AX richness differed between samples. Samples 1 and 6 did not contain any soluble AX. In addition, samples 7 and 8 contained primarily soluble AX, with the highest richness evident in these samples, at 99.2% and 90.5%, respectively. As expected, in the absence of Laminex addition, sample 3 showed the lowest WE-AX richness at 11.0%. Besides samples 7 and 8 which had very high soluble AX contents, sample 4 had the highest richness of the similarly treated samples (samples 2, 3, 4). Despite the addition of Laminex at a suboptimal temperature, this sample displayed a relatively high richness of 21.4%. In contrast, where Laminex was added to the system near the optimum temperature in sample 2, a lower richness was observed (18.4%). Even though no Laminex was added to sample 3, soluble AX was liberated (11.0%). In comparing samples 4 and 5 and the effect of grinding, sample 5 displayed a lower soluble AX richness compared to the unground sample 4; as with sample 2, losses were probable during this grinding process.

Some differences were observed in avDP, which represents the length of the AX(OS) chains; however, the magnitude of difference in those samples subjected to similar treatment processes (samples 2 to 5) was small, with the avDP ranging from 4.7 to 6.3. Interestingly, despite being produced by similar SSF processes, with sample 7 representing the supernatant fraction of sample 2, there were clear differences in the characteristics of the soluble AX component between these samples, with sample 7 having significantly higher avDP of 15.6. The avDS of the samples ranged from 0.7 to 1.3.

Apart from the activity of the added exogenous commercial enzymes, the genome of *L. plantarum* F10 was analysed for the presence genes encoding enzyme classes (hydrolases, esterases) which could have contributed to the solubilisation or utilisation of AX. From the Prokka.tbl output the presence of endo-1,4-beta xylanase (E.C 3.2.1.8) and acetyl xylan esterase (E.C 3.1.1.-) was observed. Furthermore, CAZypedia (CAZy) [42] was searched for the possible enzyme families that those enzymes of interest are within, and those were compared with results from dbCAN (those predicted as positive by more than 2 tools were considered positive) as shown in the Table 4.

**Table 4:** Enzyme classes identified in the genome of *L. plantarum* F10

Database	Enzyme class		Activity	
	CE	GH	Enzyme	Substrate
dbCAN	1, 2, 4, 9, 10	1, 2, 8, 13, 20, 25, 31, 32, 36, 38, 42, 43, 65, 73, 78, 85, 92, 125, 126	–	–
CAZypedia		3, 43, 51, 54	L-arabinofuranosidase	Hydrolysis of non-reducing α-L-arabinofuranoside residues in α-L-arabinosides
	1, 15		Feruloyl esterase	Catalyse the hydrolysis of the feruloyl group from an esterified sugar like arabinose
		8, 10, 11	Endo xylanase	Cleave (1 → 4)-*β*-d-xylosidic linkages in xylans
		30, 39, 43, 54, 116	Beta xylosidase	Hydrolysis of (1 → 4)-*β*-d-xylans, to remove xylose monomers from non-reducing end
	1,4,15		Acetyl-xylan-esterase	Catalyse hydrolysis of acetyl groups from polymeric xylan, acetylated xylose, acetylated glucose, α-naphthyl acetate

### Microbiota modulating capacity of BSG fractions

**Fig. 2:**
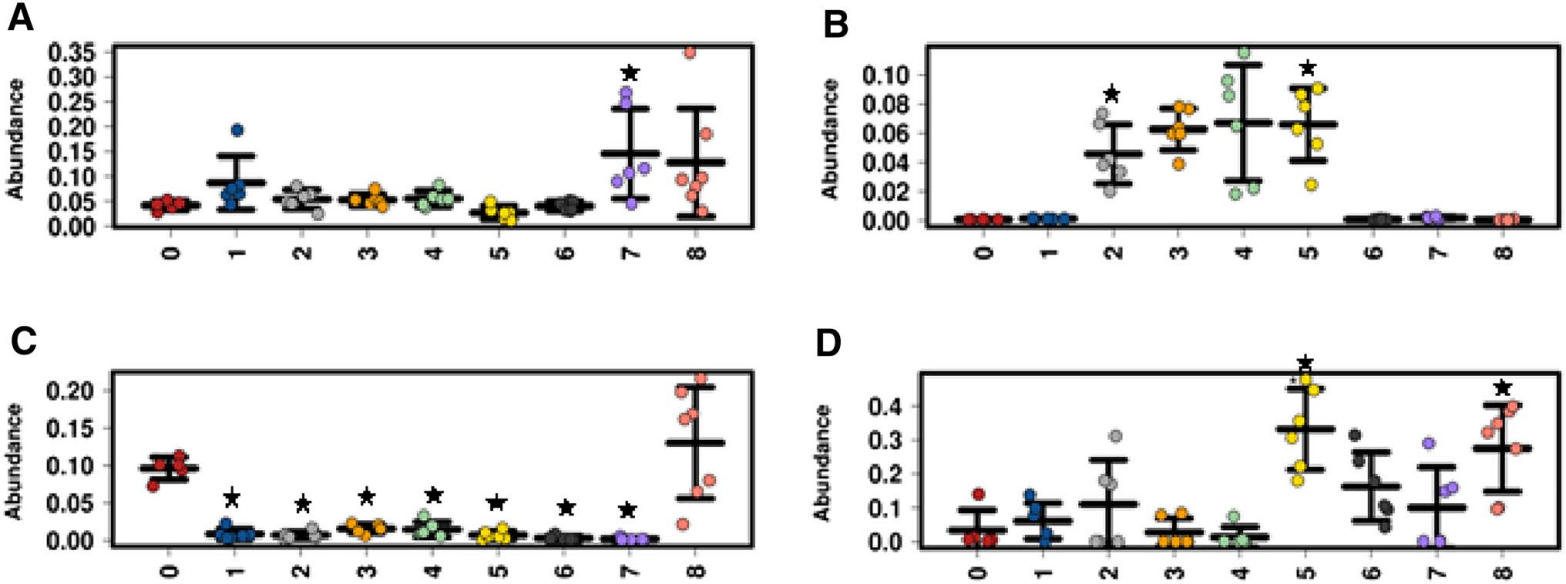
Strip plots showing the relative abundances of **a** Bifidobacteria, **b** Lactobacillus, **c** Bacteroides and **d** Shigella species for each treatment (0 = Negative control; 1 = untreated BSG; 2 = Enzyme treated, fermented, grinded before; 3 = Enzyme treated, fermented, low WE-AX yield; 4 = Enzyme treated, fermented, high WE-AX yield; 5 = Enzyme treated, fermented, high yield, grinded; 6 = Insoluble fibre extract; 7 = Soluble fibre extract; 8 = BioActor Naxus) after 24 h of fermentation (*n* = 6). The Wilcoxon rank test with *p* values adjusted for FDR by the BH correction was applied comparing each treatment (1–8) with the negative control (black star = *p* < 0.05)

**Fig. 3:**
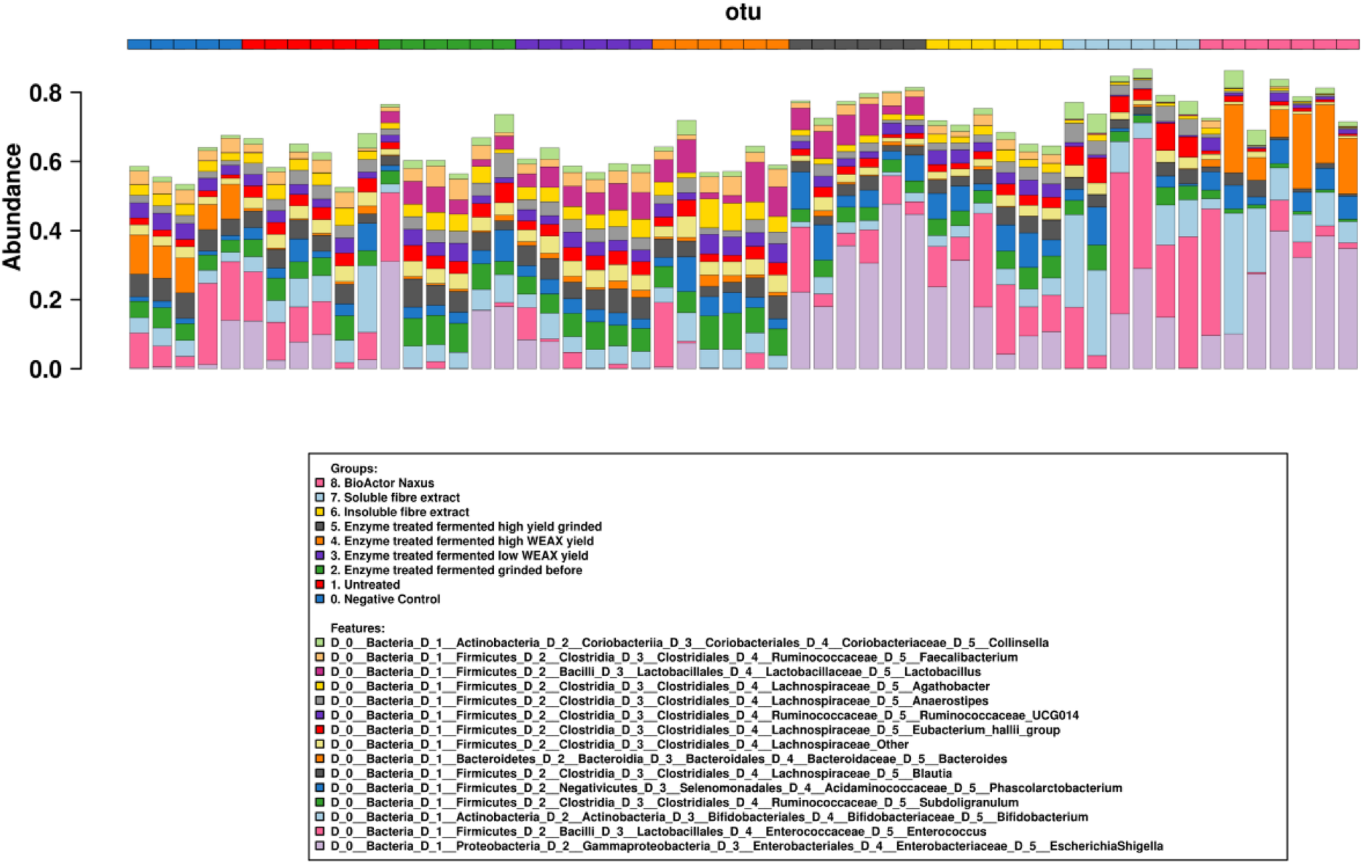
Bar chart showing the bacterial composition of the top 15 most abundant genera of each vessel after 24 h of fermentation (*n* = 6) as determined by 16S rDNA sequencing. The coloured bar on the top denotes each group as represented in the legend

**Fig. 4:**
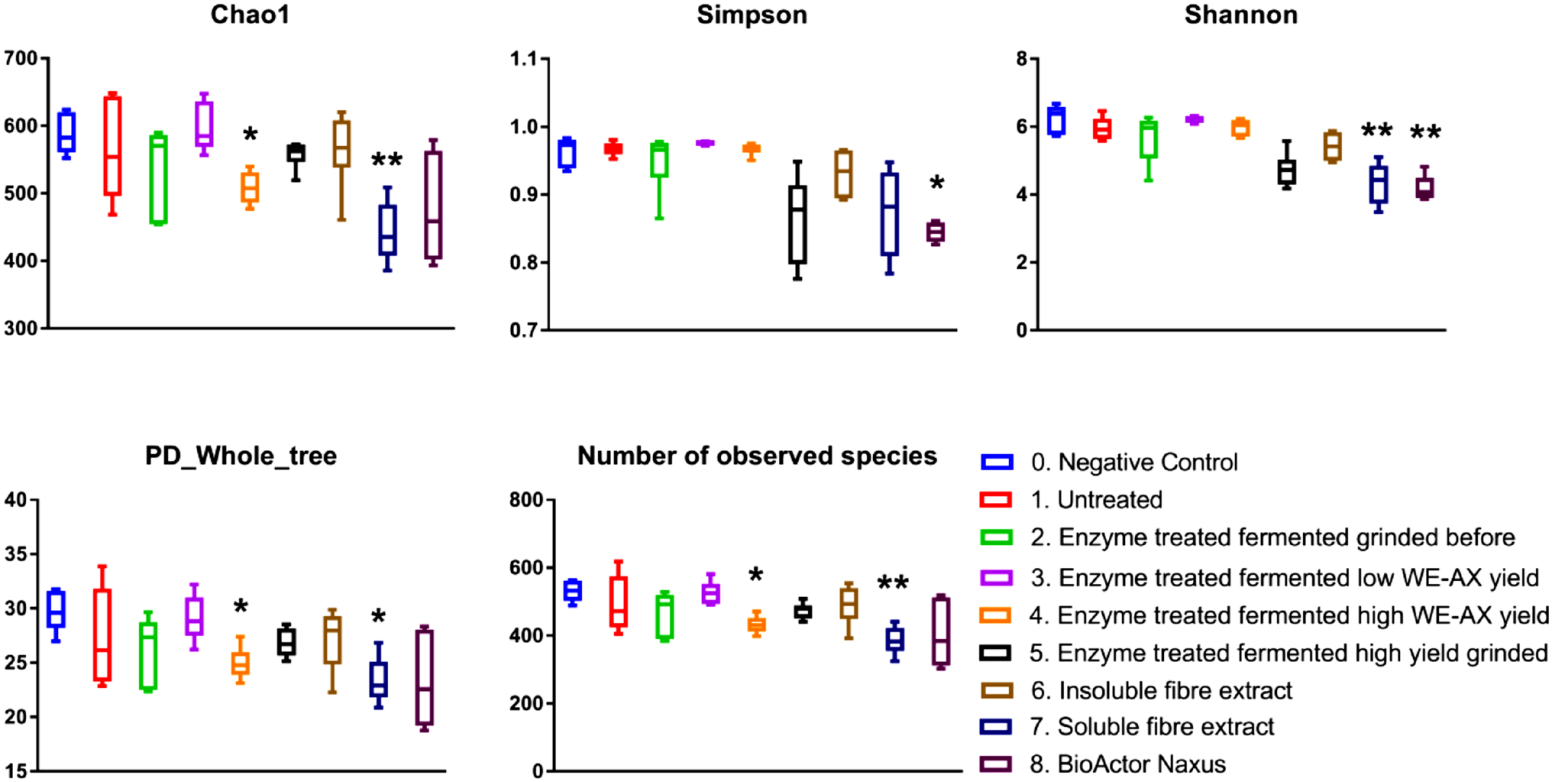
Box plot showing the different alpha diversity indices after 24 h of fermentation (*n* = 6) as determined by 16S rDNA sequencing. A one-way ANOVA with Tukey post hoc test was employed. *p < 0.05, **p < 0.01

The fermentation of sample 1 showed a significant tenfold reduction in the relative abundance of *Bacteroidetes* (*p* = 0.047) and a twofold increase in *Actinobacteria* (*p* = 0.048) at the phylum level (Supplementary Table 3), while 81 genera (Supplementary Table 4) were found to be significantly different when compared to the negative control, notably a twofold increase in *Lactobacillus* (*p* = 0.034) (Fig. 2b). A similar significant decrease in *Bacteroidetes* was found with the fermentation of samples 2–7 but no effect occurred in sample 8 (Fig. 2c & Supplementary Tables (3, 5, 6, 8, 9, 11 and 13). There were no significant differences at the genus level between sample 2 and the negative control after correcting for multiple comparisons; however, 38 genera were close to significance (*p* = 0.051). The fermentation of sample 3 yielded significant differences of 54 genera compared to the negative control (Supplementary Table 7). There was a stimulating effect of sample 3 on *Lactobacillus* relative abundance showing a 1.8-fold increase (0.13% V 0.072%; *p* = 0.035). This stimulating effect on *Lactobacillus* relative abundance was also found with sample 5 (*p* = 0.028), samples 2 and 4 showed a similar increase but was not significant after FDR correction (*p* = 0.051 & *p* = 0.05 respectively) (Fig. 2b). There were no significant differences at the genus level between sample 4 and the negative control; however, 39 genera were on the cusp of significance (*p* = 0.05) (Supplementary Table 9). Sample 5 showed a significant 8.75-fold increase in Proteobacteria (4% V 35%: *p* = 0.022) and decrease in Firmicutes (75% V 59%: *p* = 0.042) (Supplementary Table 9) while there were 88 significant differences at the genus level compared to the negative control (Supplementary Table 10). There was a significant tenfold increase in Escherichia Shigella abundances (33% V 3%, *p* = 0.022) which explains the large difference in Proteobacteria at the Phyla level (Fig. 2d). There were 63 significant differences at the genera level with sample six compared to the negative control (Supplementary Table 12). Sample 7 showed the most significant differences at the Phyla and genera level compared to negative control with 5 and 180 differences respectively (Supplementary Tables 13, 14). Sample 7 induced a 2.6-fold increase in *Lactobacillus* (7% V 19%; *p* = 0.018) (Fig. 2b) and a 3.5-fold increase in *Bifidobacterium* (4.2% V 15%; *p* = 0.035) (see Fig. 2a). Sample 8 had 115 significant different genera when compared to the negative control (Supplementary Table 16). There was a trend in increasing *Bifidobacteria* abundances with a 3.1-fold increase (4.2% V 13%: *p* = 0.051) (Fig. 2a); however, at the Phyla level sample 8 stimulated Proteobacteria abundances with a 7.25-fold increase (4% V 29%: *p* = 0.022) which can be largely explained by a stimulating effect on Escherichia Shigella abundances with a 8.2-fold increase (3.3% V 27%: *p* = 0.024) (Fig. 2d). When comparing sample 4 with 3 (high AX versus low AX) there was a significant decrease in Firmicutes (*p* = 0.022) and a significant increase in Proteobacteria (*p* = 0.025) (Supplementary Table 17) (Figs. 3, 4) When comparing samples 5 with 4 (high AX grinded Vs high AX not grinded), there were no significant differences after the BH correction.

**Fig. 5:**
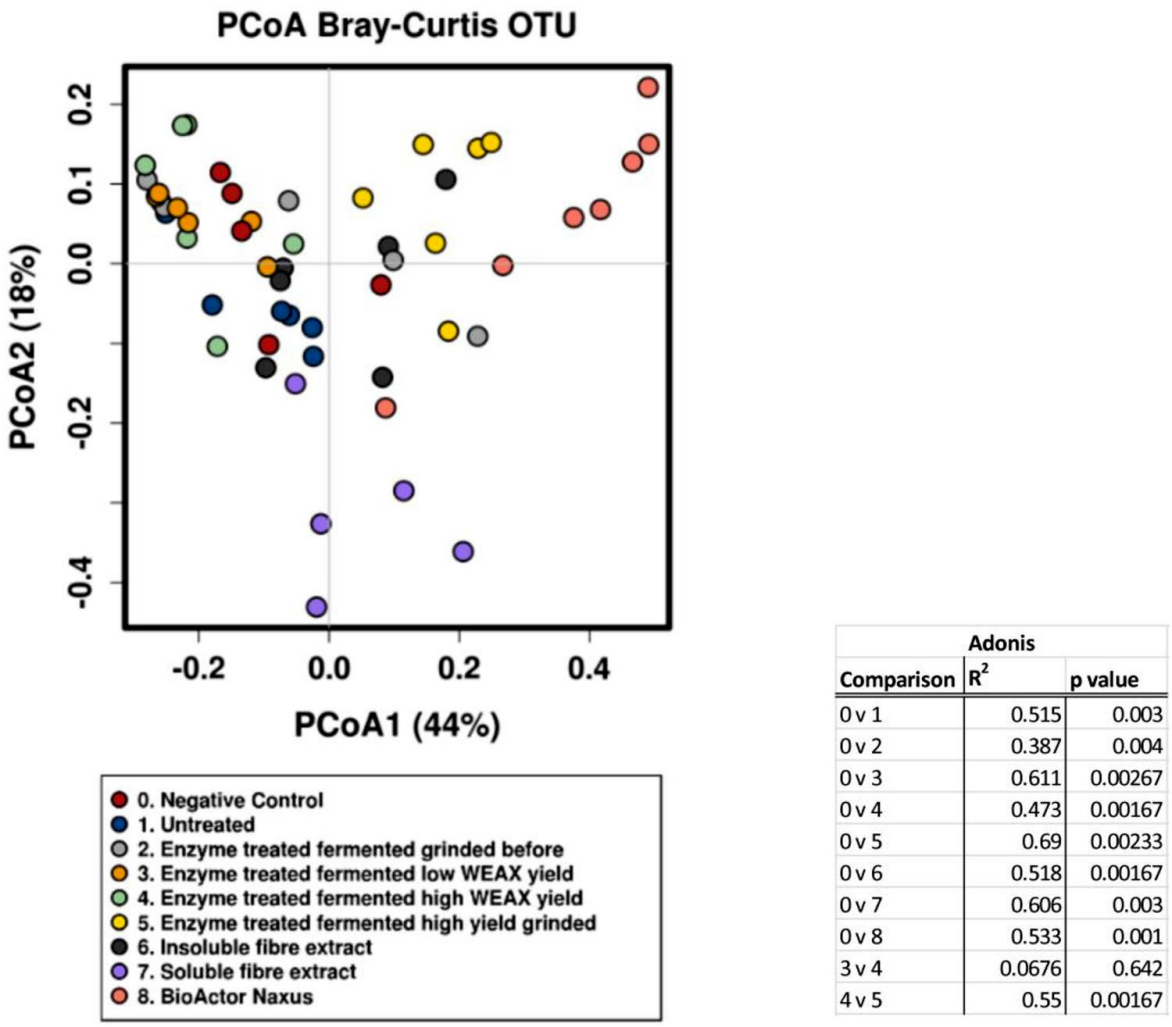
Principle coordinate analysis (PCoA) of samples using beta diversity based on Bray–Curtis operational taxonomic units (OTU) data after 24 h of fermentation (*n* = 6). Adonis dissimilarity analysis calculated from Bray–Curtis OTU distance matrices

There were a number of effects of the samples on alpha diversity when compared to the negative control (Fig. 5). Chao1 was found to be significantly decreased with samples 4 (*p* = 0.035) and 7 (*p* = 0.003), sample 8 was found to reduce the Simpson index (*p* = 0.002), samples 7 (*p* = 0.006) and 8 (*p* = 0.001) reduced the Shannon index, PD whole tree was reduced with samples 4 (*p* = 0.039) and 7 (*p* = 0.011), while the number of observed species was significantly lower with samples 4 (*p* = 0.012) and 7 (*p* = 0.002). When sample 4 was compared to sample 3, Chao1 (*p* = 0.021) and number of observed species (*p* = 0.011) were significantly lower. When sample 5 was compared to sample 4 (high AX richness versus high AX richness grinded) the Shannon index was significantly lower (*p* = 0.015).

**Fig. 6:**
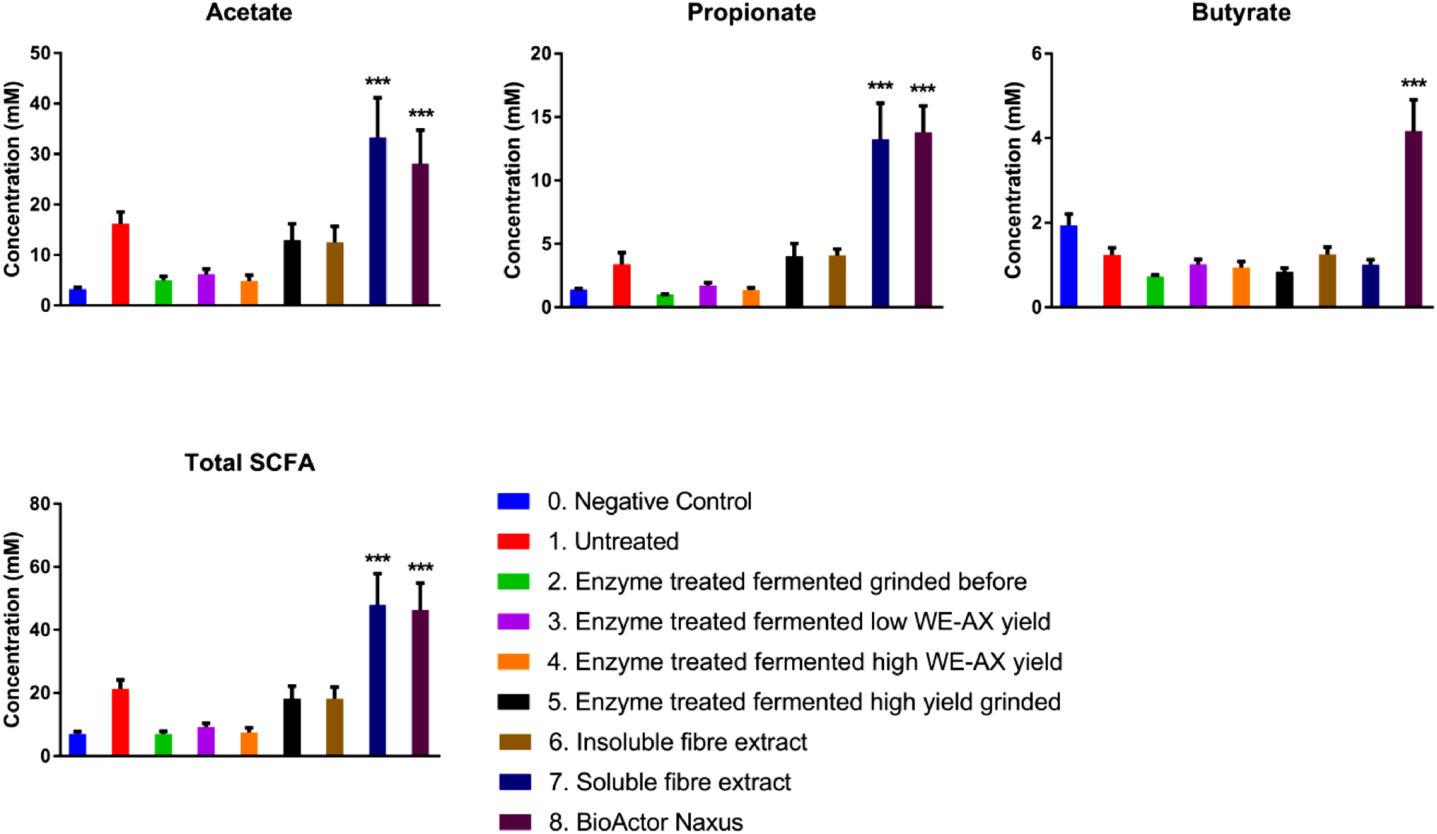
Graphs showing concentrations of acetate, propionate, butyrate and total SCFA concentrations after 24 h of fermentation (*n* = 6) as measured by GC-FID. A one-way ANOVA with Dunnett’s test—*p < 0.05, **p < 0.01, ***p < 0.001

**Fig. 7:**
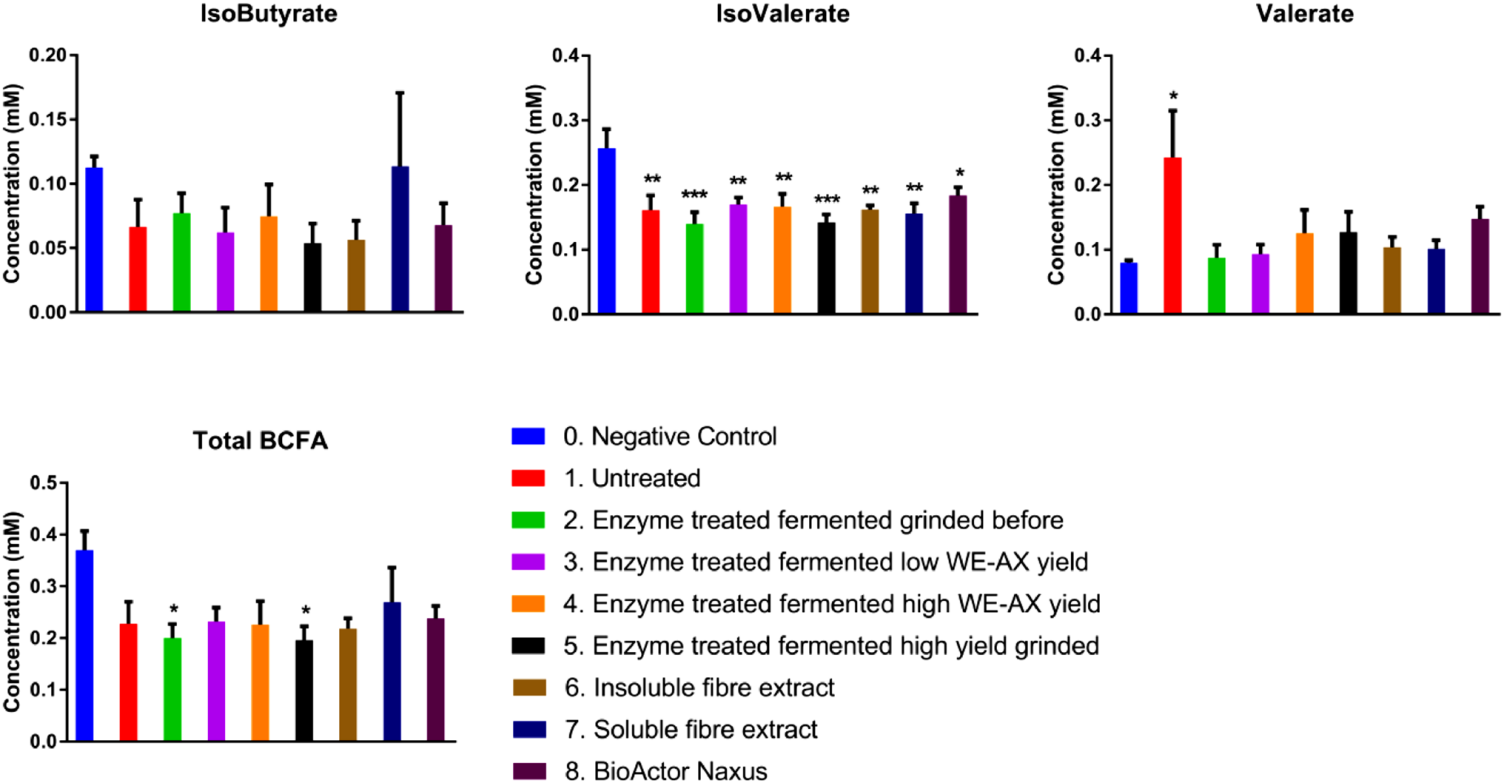
Graphs showing concentrations of isobutyrate, isovalerate, valerate and total branch chain fatty acids (BCFA) (Isobutyrate + Isovalerate) concentrations after 24 h of fermentation (*n* = 6) as measured by GC-FID. A one-way ANOVA with Dunnett’s test—*p < 0.05, **p < 0.01, ***p < 0.001

There was a significant effect of the treatments on beta diversity. The Bray–Curtis was significantly dissimilar (*r*^2^ = 0.616: *p* = 0.000333 Adonis) (Fig. 6). Samples 5, 7 and 8 appear to be the most distinct from the negative control (Fig. 6). The CCA plot reveals that all treatments separated from the negative control with sample 5 being the furthest from the negative control and sample 8 showing the greatest spread (Fig. 7).

### Short chain fatty acids

The analyses of the bacterial fermentation metabolites were carried out after 24 h of fermentation. Acetate was found to be significantly higher in sample 7 (*p* = 0.018) and sample 8 (*p* = 0.001) compared to the negative control (Fig. 8). A similar effect was found with propionate, with sample 7 (*p* < 0.001) and sample 8 (*p* < 0.001) having significantly high concentrations than the negative control (Fig. 8). No treatments had an effect of iso-butyrate production, while sample 8 was found to significantly increase butyrate production (*p* < 0.001) (Fig. 9). In contrast, there was a widespread reduction in isovalerate production compared to the control, with samples: 2 (untreated BSG, *p* = 0.005); 3 (enzyme treated, fermented, grinded before, *p* = 0.001); 4 (enzyme treated, fermented, low WE-AX richness, *p* = 0.014); 6 (insoluble fibre extract, *p* = 0.006); 7 (soluble extract, *p* = 0.003) and 8 (Naxus, *p* = 0.028) having significantly lower iso-valerate concentrations compared to the negative control (Fig. 9). The untreated BSG significantly increased valerate production (*p* = 0.024) (Fig. 9). Total SCFA concentrations were found to be significantly higher after fermentation of sample 7 (*p* = 0.005) and sample 8 (*p* < 0.001) compared to the negative control (Fig. 8). Total branch chain fatty acids (BCFA) concentrations were only significantly different with samples 2 and 5 (*p* = 0.031 and *p* = 0.029 respectively) having significantly lower total BCFA than the negative control (Fig. 9).

## Discussion

The microbiome modulating potential of BSG, a high fibre by-product of the brewing industry, was investigated with a focus on the extraction and solubilisation of potentially prebiotic AX. Owing to its high fibre and protein nature, BSG represents a potential untapped source of nutrition that to date has mainly been used as a low-value animal feed or simply sent to landfill; this latter option represents not only food loss, but loss of material that is especially high in fibre. This is important in the context of the ‘fibre gap’ which exists today in many Western nations. The nutritional composition of the BSG used in this study was comparable to other studied [6], even though the protein content was slightly higher than the typical range (15–24%) [6, 43]. The levels of arabinoxylan measured (21.9%) were also similar to previously reported values [44–47], although higher values have been reported [6, 48, 49]. The total carbohydrates likely represent mainly residual starch and the concentration are similar to starch levels reported previously [50, 51]. Total fat and ash were within expected values [6].

As with all fibres, their physicochemical characteristics effect how and the degree to which they are utilised by members of the gut microbiota. Foremost amongst these is the solubility (or insolubility) of the fibre. Many health benefits, e.g. modulation of glycaemic response, prebiotic potential, are associated with at least some level of solubility of the fibre. Other properties of the soluble fibre, such as viscosity and the extent to which the fibre is fermentable also play a role.

Notwithstanding, insoluble fibre is not undesirable, and can have a faecal bulking effect and modulate bowel regularity [52]. However, in the case of microbiota modulation as relevant to this study, insoluble fibre can be considered as being inaccessible to potentially utilising microbes.

Preliminary studies indicated a trade-off between the amount of AX solubilised and the physicochemical properties of the solubilised AX. Specifically, there was an inverse relationship between the amount of solubilised AX and the avDP of that AX. Both of these characteristics are important in determining the solubility and fermentability of the substrate. The mechanistic reason for the trade-off between soluble AX richness and avDP may be as follows: under conditions where xylanase activity is promoted a higher level of soluble AX is achieved; however, under such conditions continued enzymatic activity may result in AX(OS) already released (solubilised) being further and continuously hydrolysed to smaller OS chains thus reducing the avDP.

Sample 2 and 4 did not show significant differences in AX richness, even though the conditions under which Laminex was added in sample 2 (70 °C) were closer to the stated optimum temperature for this enzyme. Despite the use of a sub-optimal temperature in the SSF process of sample 4 (35 °C), the length of time available for enzyme action (6 h) may have still enabled the solubilisation of the majority of the accessible AX. In addition, 30% activity is retained at this temperature according to the manufacturer’s specifications. The reason that a slight but not significantly lower AX richness was achieved with sample 2 may relate to higher enzymatic activity and more hydrolysis of released AX, as discussed above. Hence, the avDP of those samples also did not showed any significant differences. A variety of studies have investigated different pre-treatment methods to aid fibre solubilisation [48, 53–56]. However, in the context of material destined for human consumption many of these methods are either non-food grade or are simply impractical in a food manufacturing setting when considering the potentially large volume of material to be processed. In this study, enzymatic methods were chosen as a means of releasing and solubilising AX(OS) from BSG. The enzymes applied were chosen because of their food-grade status and their utilisation already in the brewing industry. The authors are aware of other enzyme preparations (e.g. cellulases commonly used in second generation bioethanol production) which could have increased the efficiency of fibre solubilisation, but these were not considered for this study due to their general non-food grade status.

It has to be mentioned that, theoretically, if more of the solubilised AX is hydrolysed fully to monosaccharides then it would follow that the richness of soluble AX would decrease. Notably, sample 7 had a significantly higher avDP than the other samples that were produced with the SSF process, particularly given that the enzymatic and temperature processes used for samples 7 and 2 were similar. However, the higher avDP of sample 7 could be a consequence of the processing steps to which this sample was subjected after the SSF process i.e. concentration and drying. For example, it may be that some of the lower molecular weight oligosaccharide chains were lost during these processes thus increasing the avDP value.

The grinding process and the related reduction in particle size did not impact the avDP; however, putatively, it affected the total AX concentration due to losses of insoluble fibre. Sample 3 resulted in a lower average WE-AX richness, which was, however, not significant different from sample 2, 4 or 5, but it showed the highest avDP representing the absence of the fibre degrading enzyme.

To be considered a prebiotic, a dietary fibre must firstly be resistant to gastrointestinal digestion and absorption [57]. The samples tested appeared to be resistant to gastrointestinal digestion as the majority of the extracts remained after the in vitro digestion process, the vicious nature of the extracts did not allow the percentage retained to be calculated as there were losses when transferring between vessels.. This resistance to digestion of the samples was expected as the fibre components present in BSG would not contain significant amounts of alpha linked glucans, which are the target for the majority of the host carbohydrate-degrading digestive enzymes present in the human gastrointestinal tract. These glucans would have been released during the mashing step of the brewing process. Because all of the soluble components, including any soluble fibre will have been extracted into the wort during the brewing process, BSG is composed of almost entirely of insoluble fibre; this fibre can be thought of being ‘locked up’ in the recalcitrant lignocellulosic cell wall matrix, only a small fraction of which may be released and solubilised using a variety of chemical, physical or enzymatic means.

The microbiome modulating activity of a prebiotic can be considered to be mediated through altering the composition and metabolic activity of the gut microbiota [57, 58]. The compositional effects are defined based on the prebiotic’s selectivity. This can be summarised as stimulating putative beneficial gut bacteria species without stimulating pathobionts or pathogens. The two genera of gut-residing bacteria that have received the greatest research focus on conferring health benefits are *Bifidobacteria* and *Lactobacillus*. There are a host of health benefits attributed to these genera with evidence gleaned from *in-vitro*, animal models and human intervention studies. The stimulating effects on the important health-promoting *Lactobacillus* and *Bifidobacteria* and other resident bacteria in the gut were dependent on the characteristics of the fibre and the processing steps employed to solubilise AX. Commercial enzymes that were added for the purpose of releasing the fibre were likely the main drivers of AX solubilisation, in particular Laminex Super 3G. However, the solubilisation process involved a concomitant saccharification and fermentation with *Lactobacillus plantarum* F10. It is postulated that this fermentation aided in fibre solubilisation, firstly, by creating a more favourable pH for enzyme activity and secondly, through the release of hydrolytic enzymes. The results showed that *L. plantarum* F10 can potentially produce enzymes whose activity could have enabled further degradation of the released AX. For example, if *L. plantarum* F10 expresses L-arabinofuranosidase, arabinose would be cleaved from the arabinoxylan increasing the amount of arabinose and potentially the avDS which increases the fermentability and hence the production of SCFA. In addition, the expression of beta-xylosidase would lead to a release of xylose which influences the avDS inversely. Besides the enzyme portfolio of releasing single sugars, such as arabinose or xylose, *L. plantarum* possesses genes to express feruloyl esterase, endo-xylanase and acetyl-xylan-esterase which cleave ferulic acid, the xylan backbone and acetyl groups from arabinoxylan, respectively, and hence increase the water-extractability of arabinoxylan. Such breakdown could increase the fermentability of the resulting products by other *Lactobacillus* species in the environment. This phenomenon of cross feeding amongst *Lactobacillus* species has been shown to occur with other prebiotics, such as inulin type fructans [59].

Few studies have found increases in *Lactobacillus* with AX or AX-rich foods in a high-fat diet rat study [60], in two piglet feeding trials with AX from wheat (Naxus) [10, 61], and in a double blinded, placebo-controlled, crossover human intervention study with AX-rich breakfast cereal intervention [62]. In contrast, another human intervention study consisting of wheat bran AX consumption showed no effect and even a decrease in *Lactobacillus* levels [63]. However, the specific characteristics of the fibre may have played a role. It is noteworthy given the *L. plantarum* F10 fermentation strain used in this study, that this species was previously observed as the dominant *Lactobacillus* found in a study comparing AX (Naxus) and inulin in an *in-vitro* gut model (SHIME) [64]. Similarly, increases in lactobacilli (two–fourfold) were reported by Sanchez et al. in a SHIME model [65]. Many of the samples in the current study induced an increase in the relative abundance in lactobacilli (approx. twofold), including sample 1 (untreated BSG) and sample 3 (no Laminex addition). The greatest increase was observed with the soluble AX extract (sample 7) at 2.6-fold.

Despite the above variable effects on *Lactobacillus* levels, the bifidogenic effect of AX(OS) and the effects of AX enrichment on this genus are far less ambiguous, with numerous studies showing a stimulating effect on *Bifidobacteria* populations. Such positive effects have been observed in pure culture [66, 67], *in-vitro* batch and gut model fermentations [8, 9, 64, 65, 68, 69], *in-vivo* animal models [9, 41, 70–72] and in human intervention studies [9, 62, 63, 73–76], independent of factors such fibre physicochemical characteristics and dosage or length of the treatment time. Given the grist composition from which the BSG used in this study was produced, consisting of barely and rice, it is important to consider the characteristics of the AX from these cereals. Both barley and rice consumption was found to increase *Bifidobacteria* in humans [77]. Rice bran has been found to increase *Bifidobacteria* in healthy adults [78]. Rumpagaporn et al. (2015) showed that AX extracted from different cereal brans fermented at different rates in faecal slurry batch fermentation. Wheat and corn AXs were characterised as slow fermenting while rice and sorghum AXs were found to be fast fermenting. The difference in fermentation rates was linked to the varied physicochemical characteristics of the AXs from the different cereals [79]. A similar finding was observed by Tuncil et al. (2017) who observed that more-complex arabinoxylan structures tended to be degraded more slowly by the colonic microbiota [80]. Reis et al. used two different processing methods (ultrasonic and alkaline extraction) to extract AX from BSG. The resulting two AX fractions were fermented at the different rates, with one showing a greater bifidogenic effect than the other [69]. This demonstrates that even the extraction method can influence the fermentation of the fibre, likely because of different resulting fibre physicochemical characteristics. Interestingly, a bifidogenic effect was only found in the soluble fibre extract (sample 7), and a close to significant effect with the Naxus, suggesting that solubility of the fibres plays the key role. The lack of a bifidogenic effect seen with the less soluble extracts suggests that the fibre components are inaccessible to the *Bifidobacteria*. The distinct chemical characteristics of these samples in terms of higher soluble AX content and higher avDP when compared to the other samples was likely linked to these bifidogenic effects. No bifidogenic effect was observed for those samples that had a significantly lower avDP (samples 2 through 5). Interestingly, studies have previously shown low molecular weight AX of similar avDP (i.e. approx. avDP 5) to have a bifidogenic effect. However, other characteristics of the AXOS preparation (e.g. avDS) and the design of the study were different, making direct comparison of results difficult [70, 73]. In addition, the levels of soluble AX in sample 2 through 6 was significantly lower than sample 7 or 8; thus, levels may have been insufficient to promote a bifidogenic effect [70]. The AX extracted in all samples had a high avDS (A/X) ratio, greater than 0.6. This suggests a high level of arabinose substitution on the xylose backbone. Higher arabinose substitutions have also been associated with stimulation of *Lactobacillus* as members that can preferentially utilise this monosaccharide. This may explain the stimulatory effect of most samples on this genus. Analysis of *B. longum* strains also revealed preferential utilisation of arabinose side, but utilisation of xylose was not efficient. It was suggested that *B. longum* produces one or more α-L-arabinofuranosidases able to cleave the arabinose side-chains from arabinoxylan but does not produce Signiant β-D-xylanase to hydrolyse the xylan backbone and make this available for fermentation [81]. Indeed, AX(OS) degradation by bifidobacteria has been shown to be strain dependent, with initial consumption of arabinose, with or without subsequent xylose backbone utilisation [82].

Both *in-vivo* and *in-vitro* studies have demonstrated the prebiotic potential of wheat AX(OS) [8, 64, 83]. The production of short chain fatty acids (SCFAs) by the gut microbiota as a consequence of fibre utilisation is an important mechanism through which fibre consumption is linked to consequent health benefits for the host. The SCFA data indicate that the soluble fibre extract (sample 7) and the Naxus were the most fermentable of the tested substrates as both were found to significantly increase total SCFA concentrations. The soluble fibre extract also significantly increased acetate and propionate (Fig. 7). The increased propionate production could be formed through the Acrylate pathway by stimulated members of the *Lachnospiraceae* family; however, neither succinate nor lactate were measured in this study to confirm this assumption. Acetate has been linked with anti-obesogenic [84], anti-inflammatory [85] and neuroprotective bioactivities [86]. It is the most abundant bacterial-derived SCFA in the blood with up to 40% of colonic-derived acetate entering systemic circulation [87] and therefore may have greater extra-intestinal effects than other SCFAs. It can be used as an energy source by astrocytes in the brain [88] as well as hypothalamic neuronal activation to induce satiety [84]. In the liver, it is involved in cholesterol and lipid metabolism [89, 90]. Propionate also has a number of bioactivities attributed to it such as anti-inflammatory [91, 92], anti-cancer [93] and anti-lipogenic effects [94, 95]. It can be used as a substrate for gluconeogenesis [96] and can induce the expression of GLP-1, PYY [97], and leptin [98]. It is estimated that around 10% of colonic-derived propionate may enter systemic circulation as the majority is used by the liver after transfer from the gut through the hepatic portal vein [87].

The increased acetate and propionate in the soluble fibre extract (sample 7) correlate with those of previous studies examining the prebiotic potential of AX [58]. Of these studies, many have observed increased concentrations of both acetate and propionate, independent of AX characteristic or model [83, 99, 100], or increased propionate alone [8, 10, 61, 79]. The increased acetate production is likely linked to the increased *Bifidobacteria* levels observed as acetate, along with lactate, are major fermentation products of this group [99]. Propionate production has been linked to the *Bacteroides-Prevotella* group [68]. Butyrate production appears to be more variable; comparatively fewer studies showed a change or increase in the levels of this SCFA alongside acetate and propionate [9, 69, 83, 101, 102]. None of the samples tested in the current study led to changes in butyrate levels. This may be owing to the butyrigenic components being removed with the wort, during the dialysis step or that the bacteria responsible were not surviving in the model of this study. Weckx et al. (2015), suggested that cross feeding of *Eubacterium rectale* from *Bifidobacteria* derived acetate could explain the bifidogenic effect of AXOS [103]. The results of this study suggest that another pathway such as the butyrate kinase pathway may dominate during Naxus fermentation as acetate was significantly increased in both the soluble extract and Naxus, but only Naxus fermentation resulted in increased butyrate production. Although not significant, a trend in decreasing total BCFA, as has been seen in previous studies, was observed [10, 70]. Branch chain fatty acids originate from the breakdown of protein, which is considered undesirable [104]. All samples lead to significantly reduced isovalerate production.

The two substrates (soluble fibre extract & Naxus) that showed the largest increase in fermentation as assessed by increased total SCFA production also had significant effects on several indices of alpha diversity. However, these effects resulted in significantly reduced alpha diversity scores. This phenomenon may be explained by the substrates stimulating a limited number of bacterial species which would drive down the alpha diversity. This effect on a limited amount of species could be explained by the limited number of glycosidic bounds found within these substrates. The reduced alpha diversity is a weakness of this model as it would be the inverse of what would be expected to occur in vivo*.* Other factors such as the lack of host microbe interactions and no preservation of the mucus layer which would occur in vivo may also help explain these reduced alpha diversities.

## Conclusion

All AX samples derived from BSG induced microbial shifts in the faecal microbiota model used. The highly substituted nature of the AX likely was the reason for increased *Lactobacillus* levels. One BSG-derived sample in particular, the soluble fibre extract (sample 7), led to a significant increase in *Bifidobacteria*, comparable to Naxus, an AX fibre well studied for its prebiotic effects. Fermentation of this soluble fibre extract lead to significant increases in acetate and propionate, both of which are associated with positive health outcomes for the host. The reason for a lack of a bifidogenic effect with many of the samples may relate to the significantly lower avDP of the AX extracted from these samples, or the significantly lower amount of AX that was solubilised. Conversely, the higher avDP and higher soluble AX concentration in the soluble fibre extract may have promoted the observed bifidogenic effect. Thus, the extraction of AX from BSG can yield a functional fibre with potential prebiotic properties similar to AX from other cereals. However, factors such as the concentration of the solubilised fibre and its molecular characteristics are critical. A future challenge will be the scale up of the process that enables the large-scale extraction of AX from BSG that demonstrates the desired physicochemical characteristics.

## Supplementary Information

Below is the link to the electronic supplementary material.

Supplementary file1 (DOCX 109 KB)
