# Supplementary material for: Extraction and characterisation of arabinoxylan from brewers spent grain and investigation of microbiome modulation potential
Source: Eur J Nutr. 2021 May 31;60(8):4393–411. doi: 10.1007/s00394-021-02570-8 (PMC8572209; doi:10.1007/s00394-021-02570-8)
Supplement: Supplementary file 1 — Supplementary file1 (DOCX 109 KB) [file 394_2021_2570_MOESM1_ESM.docx]

Supplementary table 1: Composition of the mobile phases used for HPAE-PAD.

| **Isocratic method on CarboPac PA1*^a^*** | | | | |
| --- | --- | --- | --- | --- |
| **time [min]** | A*^b^* [%] | B*^c^* [%] | C*^d^* [%] |  |
| **0** | 91.9 | 8.1 | 0 | separation of  analytes |
| **25** | 91.9 | 8.1 | 0 |  |
| **26** | 0 | 0 | 100 | column cleanup |
| **31** | 0 | 0 | 100 |  |
| **32** | 0 | 100 | 0 | regeneration gold surface |
| **47** | 0 | 100 | 0 |  |
| **48** | 91.9 | 8.1 | 0 | re-equilibration |
| **60** | 91.9 | 8.1 | 0 |  |
| *^a^* flow rate 0.25 ml/min, column temperature 25 °C.  *^b^* purified water.  *^c^* 225 mM NaOH.  *^d^* 500 mM NaOAc. | | | | |

Supplementary table 2: Preparation of simulated digestion fluids

| **Simulated Fluid** | **Constituents ^a^** |
| --- | --- |
| Simulated Salivary Fluid (SSF), pH 7.0* | 15.1mM KCl ; 3.7mM KH_2_PO_4_; 13.6mM NaHCO_3_; 0.15mM MgCl_2_(H_2_O)_6_; 0.06mM (NH_4_)_2_CO_3_ |
| Simulated Gastric Fluid (SGF), pH 3.0* | 6.9mM KCl; 0.9mM KH_2_PO_4_; 25mM NaHCO_3_; 47.2mM NaCl; 0.1mM MgCl_2_(H_2_O)_6_; 0.5mM (NH_4_)_2_CO_3_ |
| Simulated Intestinal Fluid (SIF), pH 7.0* | 6.8mM KCl ; 0.8mM KH_2_PO_4_; 85mM NaHCO_3_; 38.4mM NaCl; 0.33mM MgCl_2_(H_2_O)_6_ |

*pH adjusted where necessary with 2M HCl.

^a^ CaCl_2_(H_2_O)_2_ added to the final mixture of simulated digestion fluids and extract to prevent precipitation in electrolyte stock solutions.

Supplementary table 3. Table showing Wilcoxon rank p values, BH corrected p values, means, medians and fold changes (means) of significant phyla of 1.BSG treated vessels compared to 0.negative control treated vessels after 24 hours of fermentation.

| **Taxa.__OTU___** | **P (rank test)** | **BH corrected** | **0. Negative Control mean** | **1. Untreated mean** | **0. Negative Control median** | **1. Untreated median** | **Fold Change** |
| --- | --- | --- | --- | --- | --- | --- | --- |
| Bacteroidetes | 0.0043 | 0.047 | 0.14 | 0.014 | 0.14 | 0.012 | -10.21 |
| Actinobacteria | 0.0087 | 0.048 | 0.059 | 0.12 | 0.062 | 0.098 | 2.021 |

Supplementary table 4. Table showing Wilcoxon rank p values, BH corrected p values, means, medians and fold changes (means) of significant genera of BSG treated vessels and negative control treated vessels after 24 hours of fermentation.

| **Taxa.__OTU___** | **P (rank test)** | **BH corrected** | **0. Negative Control mean** | **1. Untreated mean** | **0. Negative Control median** | **1. Untreated median** | **Fold Change** |
| --- | --- | --- | --- | --- | --- | --- | --- |
| Veillonella | 0.0043 | 0.034 | 0.00027 | 0.00079 | 0.00026 | 0.00072 | 2.97 |
| Dialister | 0.0043 | 0.034 | 0.0078 | 0.024 | 0.0087 | 0.024 | 3.137 |
| Turicibacter | 0.0043 | 0.034 | 0.0019 | 0.003 | 0.0021 | 0.0031 | 1.625 |
| Erysipelotrichaceae_UCG003 | 0.0043 | 0.034 | 0.011 | 0.03 | 0.011 | 0.027 | 2.759 |
| Coprobacillus | 0.0043 | 0.034 | 0.00043 | 0.00016 | 0.00039 | 0.00016 | -2.662 |
| Ruminococcaceae__UBA1819 | 0.0043 | 0.034 | 0.00096 | 0.0003 | 0.001 | 0.00028 | -3.218 |
| Ruminococcus_2 | 0.0043 | 0.034 | 0.034 | 0.016 | 0.032 | 0.015 | -2.095 |
| Candidatus_Soleaferrea | 0.0043 | 0.034 | 0.0011 | 0.000088 | 0.0012 | 0.000085 | -12.84 |
| Peptostreptococcaceae_uncultured | 0.0043 | 0.034 | 0.0039 | 0.0058 | 0.0038 | 0.0058 | 1.464 |
| Peptostreptococcaceae_Romboutsia | 0.0043 | 0.034 | 0.006 | 0.0082 | 0.0059 | 0.0084 | 1.352 |
| Tyzzerella_3 | 0.0043 | 0.034 | 0.002 | 0.00031 | 0.0021 | 0.00032 | -6.391 |
| Lachnospiraceae_ND3007_group | 0.0043 | 0.034 | 0.0083 | 0.0024 | 0.0089 | 0.0018 | -3.452 |
| Eubacterium_hallii_group | 0.0043 | 0.034 | 0.018 | 0.036 | 0.019 | 0.035 | 2.011 |
| Anaerostipes | 0.0043 | 0.034 | 0.02 | 0.037 | 0.022 | 0.037 | 1.813 |
| Christensenellaceae_uncultured | 0.0043 | 0.034 | 0.00079 | 0.0004 | 0.00082 | 0.00038 | -1.985 |
| Christensenellaceae_R7_group | 0.0043 | 0.034 | 0.03 | 0.013 | 0.029 | 0.011 | -2.361 |
| Leuconostoc | 0.0043 | 0.034 | 0.0009 | 0.0029 | 0.00095 | 0.0029 | 3.287 |
| Lactobacillus | 0.0043 | 0.034 | 0.00072 | 0.0013 | 0.00066 | 0.0013 | 1.826 |
| Carnobacterium | 0.0043 | 0.034 | 0.00032 | 0.031 | 0.00029 | 0.036 | 97.73 |
| Parabacteroides | 0.0043 | 0.034 | 0.022 | 0.00029 | 0.023 | 0.00024 | -73.85 |
| Rikenellaceae_RC9_gut_group | 0.0043 | 0.034 | 0.001 | 0.000098 | 0.00098 | 0.000075 | -10.15 |
| Alistipes | 0.0043 | 0.034 | 0.011 | 0.0017 | 0.012 | 0.0017 | -6.72 |
| Muribaculaceae_uncultured_bacterium | 0.0043 | 0.034 | 0.0036 | 0.00066 | 0.0037 | 0.0007 | -5.43 |
| Barnesiella | 0.0043 | 0.034 | 0.0032 | 0.00033 | 0.0025 | 0.0003 | -9.666 |
| Sanguibacter | 0.005 | 0.034 | 0 | 0.000057 | 0 | 0.00005 | 56670 |
| Exiguobacterium | 0.0054 | 0.034 | 0 | 0.00011 | 0 | 0.0001 | 111700 |
| Frigoribacterium | 0.0054 | 0.034 | 0 | 0.00018 | 0 | 0.00017 | 175000 |
| Rhodococcus | 0.0054 | 0.034 | 0 | 0.00028 | 0 | 0.00026 | 275000 |
| AllorhizobiumNeorhizobiumPararhizobiumRhizobium | 0.0055 | 0.034 | 0 | 0.00015 | 0 | 0.00014 | 151700 |
| Clostridium_sensu_stricto_13 | 0.0055 | 0.034 | 0.0098 | 6.7E-06 | 0.0012 | 0 | -1472 |
| Glutamicibacter | 0.0055 | 0.034 | 0 | 0.00099 | 0 | 0.00092 | 990000 |
| Arthrobacter | 0.0055 | 0.034 | 0 | 0.00028 | 0 | 0.00029 | 283300 |
| Rathayibacter | 0.0055 | 0.034 | 0 | 0.0001 | 0 | 0.00011 | 101700 |
| Leucobacter | 0.0055 | 0.034 | 0 | 0.00017 | 0 | 0.00016 | 173300 |
| Clavibacter | 0.0055 | 0.034 | 0 | 0.00041 | 0 | 0.00042 | 410000 |
| Corynebacterium_1 | 0.0055 | 0.034 | 0 | 0.0017 | 0 | 0.0015 | 1687000 |
| Finegoldia | 0.0067 | 0.034 | 0.000078 | 8.3E-06 | 0.00008 | 0 | -9.359 |
| Prevotella_7 | 0.0067 | 0.034 | 0.000002 | 0.00018 | 0 | 0.00018 | 89.12 |
| Peptoniphilus | 0.0074 | 0.034 | 0.0049 | 0.000023 | 0.0027 | 0.000025 | -210.1 |
| Anaerococcus | 0.0075 | 0.034 | 0.001 | 0.000043 | 0.00087 | 0.00004 | -24.09 |
| Butyrivibrio | 0.0078 | 0.034 | 0.00041 | 0.000093 | 0.00046 | 0.00008 | -4.414 |
| Butyricimonas | 0.0078 | 0.034 | 0.00048 | 0.000038 | 0.00029 | 0.00002 | -12.42 |
| Haemophilus | 0.008 | 0.034 | 0.00025 | 0.00002 | 0.0002 | 0.000015 | -12.3 |
| Bilophila | 0.008 | 0.034 | 0.0029 | 0.00081 | 0.0026 | 0.00073 | -3.568 |
| Holdemania | 0.008 | 0.034 | 0.00024 | 0.000082 | 0.00025 | 0.0001 | -2.914 |
| Faecalitalea | 0.008 | 0.034 | 0.00034 | 0.00005 | 0.00035 | 0.00005 | -6.84 |
| Ruminiclostridium_1 | 0.008 | 0.034 | 0.0013 | 0.00024 | 0.0012 | 0.00027 | -5.617 |
| Lachnospiraceae_UCG003 | 0.008 | 0.034 | 0.0021 | 0.0001 | 0.002 | 0.000055 | -19.9 |
| Clostridiales_vadinBB60_group_uncultured_bacterium | 0.008 | 0.034 | 0.0002 | 0.000033 | 0.00019 | 0.00003 | -6.12 |
| Lactococcus | 0.008 | 0.034 | 0.00016 | 0.00058 | 0.00017 | 0.00058 | 3.615 |
| Weissella | 0.008 | 0.034 | 0.00027 | 0.00077 | 0.00027 | 0.00069 | 2.816 |
| Prevotella_9 | 0.008 | 0.034 | 0.000024 | 0.00055 | 0.00002 | 0.0003 | 22.78 |
| Bacteroides | 0.008 | 0.034 | 0.096 | 0.0085 | 0.1 | 0.0069 | -11.26 |
| Actinomyces | 0.008 | 0.034 | 0.00012 | 0.00031 | 0.0001 | 0.00029 | 2.599 |
| Phascolarctobacterium | 0.0087 | 0.034 | 0.014 | 0.043 | 0.013 | 0.034 | 3.051 |
| Paeniclostridium | 0.0087 | 0.034 | 0.00022 | 0.00042 | 0.00021 | 0.00041 | 1.904 |
| Lachnospiraceae_Other | 0.0087 | 0.034 | 0.026 | 0.039 | 0.027 | 0.04 | 1.526 |
| Lachnospiraceae_uncultured | 0.0087 | 0.034 | 0.0029 | 0.0048 | 0.0026 | 0.0044 | 1.666 |
| Lachnoclostridium | 0.0087 | 0.034 | 0.012 | 0.02 | 0.013 | 0.02 | 1.659 |
| Peptostreptococcus | 0.009 | 0.034 | 0.000026 | 1.7E-06 | 0.00002 | 0 | -15.59 |
| Clostridia_DTU014_uncultured_bacterium | 0.0096 | 0.036 | 0.00014 | 8.3E-06 | 0.00018 | 0.000005 | -17.04 |
| Peptococcaceae_uncultured | 0.01 | 0.037 | 0.00032 | 0.00017 | 0.00029 | 0.00018 | -1.925 |
| Erysipelotrichaceae_UCG007 | 0.012 | 0.044 | 0.000004 | 0.000032 | 0 | 0.00003 | 7.915 |
| Ezakiella | 0.013 | 0.046 | 0.00011 | 0.000017 | 0.0001 | 0.000015 | -6.36 |
| Senegalimassilia | 0.013 | 0.046 | 0.00052 | 0.00084 | 0.00056 | 0.0008 | 1.618 |
| Mollicutes_RF39_Ambiguous_taxa | 0.015 | 0.048 | 0.000014 | 0 | 0.00001 | 0 | -14000 |
| Lachnospiraceae_GCA900066755 | 0.015 | 0.048 | 0.000016 | 0 | 0.00001 | 0 | -16000 |
| Clostridiales_Family_XIII_S5A14a | 0.015 | 0.048 | 0.00003 | 0 | 0.00003 | 0 | -30000 |
| Helcococcus | 0.015 | 0.048 | 0.000046 | 0 | 0.00006 | 0 | -46000 |
| Gallicola | 0.015 | 0.048 | 0.000038 | 0 | 0.00004 | 0 | -38000 |
| Enterobacteriaceae_Other | 0.016 | 0.048 | 0.000006 | 0.00066 | 0 | 0.00046 | 109.4 |
| Bacillus | 0.016 | 0.048 | 0 | 0.000022 | 0 | 0.00002 | 21670 |
| Clostridium_innocuum_group | 0.017 | 0.048 | 0.00016 | 0.000068 | 0.00014 | 0.00007 | -2.283 |
| Ruminococcaceae_uncultured | 0.017 | 0.048 | 0.0091 | 0.0047 | 0.01 | 0.0044 | -1.939 |
| Ruminococcaceae_UCG004 | 0.017 | 0.048 | 0.00033 | 0.00064 | 0.00032 | 0.00058 | 1.933 |
| Ruminococcaceae_UCG003 | 0.017 | 0.048 | 0.0029 | 0.00094 | 0.003 | 0.00058 | -3.095 |
| Ruminococcaceae_UCG002 | 0.017 | 0.048 | 0.029 | 0.015 | 0.029 | 0.014 | -1.936 |
| Ruminiclostridium_5 | 0.017 | 0.048 | 0.0034 | 0.0021 | 0.0036 | 0.0019 | -1.655 |
| Peptococcus | 0.017 | 0.048 | 0.00038 | 0.000043 | 0.00025 | 0.000045 | -8.677 |
| Lachnospiraceae_FCS020_group | 0.017 | 0.048 | 0.0019 | 0.001 | 0.0019 | 0.00088 | -1.826 |
| Coprococcus_2 | 0.017 | 0.048 | 0.0097 | 0.018 | 0.0097 | 0.019 | 1.833 |

Supplementary table 5. Table showing Wilcoxon rank p values, BH corrected p values, means, medians and fold changes (means) of significant phyla of 2.Enzyme treated fermented grinded before treated vessels and 0.negative control treated vessels after 24 hours of fermentation.

| **Taxa.__OTU___** | **P (rank test)** | **BH corrected** | **0. Negative Control mean** | **2. Enzyme treated fermented grinded before mean** | **0. Negative Control median** | **2. Enzyme treated fermented grinded before median** | **Fold Change** |
| --- | --- | --- | --- | --- | --- | --- | --- |
| Bacteroidetes | 0.0043 | 0.043 | 0.14 | 0.012 | 0.14 | 0.011 | 12.04 |

Supplementary table 6. Table showing Wilcoxon rank p values, BH corrected p values, means, medians and fold changes (means) of significant phyla of 3. Enzyme treated fermented low WEAX yield treated vessels and 0.negative control treated vessels after 24 hours of fermentation.

| **Taxa.__OTU___** | **P (rank test)** | **BH corrected** | **0. Negative Control mean** | **3. Enzyme treated fermented low WEAX yield mean** | **0. Negative Control median** | **3. Enzyme treated fermented low WEAX yield median** | **Fold Change** |
| --- | --- | --- | --- | --- | --- | --- | --- |
| Bacteroidetes | 0.0043 | 0.043 | 0.14 | 0.028 | 0.14 | 0.029 | -4.971 |

Supplementary table 7. Table showing Wilcoxon rank p values, BH corrected p values, means, medians and fold changes (means) of significant genera of 3. Enzyme treated fermented low WEAX yield treated vessels and 0.negative control treated vessels after 24 hours of fermentation.

| **Taxa.__OTU___** | **P (rank test)** | **BH corrected** | **0. Negative Control mean** | **3. Enzyme treated fermented low WEAX yield mean** | **0. Negative Control median** | **3. Enzyme treated fermented low WEAX yield median** | **Fold Change** |
| --- | --- | --- | --- | --- | --- | --- | --- |
| Sutterella | 0.0043 | 0.035 | 0.000048 | 0.0019 | 0.00004 | 0.0013 | 40.24 |
| Desulfovibrio | 0.0043 | 0.035 | 0.00066 | 0.0012 | 0.00075 | 0.0012 | 1.892 |
| Veillonella | 0.0043 | 0.035 | 0.00027 | 0.00089 | 0.00026 | 0.00094 | 3.346 |
| Dialister | 0.0043 | 0.035 | 0.0078 | 0.027 | 0.0087 | 0.026 | 3.467 |
| Phascolarctobacterium | 0.0043 | 0.035 | 0.014 | 0.036 | 0.013 | 0.036 | 2.52 |
| Erysipelotrichaceae_UCG003 | 0.0043 | 0.035 | 0.011 | 0.023 | 0.011 | 0.023 | 2.114 |
| Coprobacillus | 0.0043 | 0.035 | 0.00043 | 0.00016 | 0.00039 | 0.00015 | -2.635 |
| Ruminococcus_2 | 0.0043 | 0.035 | 0.034 | 0.015 | 0.032 | 0.015 | -2.215 |
| Ruminococcus_1 | 0.0043 | 0.035 | 0.0035 | 0.0064 | 0.0035 | 0.0069 | 1.839 |
| Ruminococcaceae_UCG004 | 0.0043 | 0.035 | 0.00033 | 0.00075 | 0.00032 | 0.00072 | 2.254 |
| Ruminococcaceae_UCG003 | 0.0043 | 0.035 | 0.0029 | 0.0012 | 0.003 | 0.0013 | -2.342 |
| Candidatus_Soleaferrea | 0.0043 | 0.035 | 0.0011 | 0.000082 | 0.0012 | 0.000075 | -13.89 |
| Butyricicoccus | 0.0043 | 0.035 | 0.0015 | 0.0034 | 0.0016 | 0.0035 | 2.256 |
| Lachnospiraceae_Other | 0.0043 | 0.035 | 0.026 | 0.045 | 0.027 | 0.046 | 1.767 |
| Lachnospiraceae_uncultured | 0.0043 | 0.035 | 0.0029 | 0.0054 | 0.0026 | 0.0052 | 1.859 |
| Tyzzerella_3 | 0.0043 | 0.035 | 0.002 | 0.00044 | 0.0021 | 0.00044 | -4.438 |
| Lachnospiraceae_UCG003 | 0.0043 | 0.035 | 0.0021 | 0.00012 | 0.002 | 0.00012 | -17.66 |
| Lachnospiraceae_ND3007_group | 0.0043 | 0.035 | 0.0083 | 0.0032 | 0.0089 | 0.0033 | -2.595 |
| Lachnoclostridium | 0.0043 | 0.035 | 0.012 | 0.024 | 0.013 | 0.025 | 2.046 |
| Fusicatenibacter | 0.0043 | 0.035 | 0.015 | 0.02 | 0.015 | 0.02 | 1.377 |
| Eubacterium_hallii_group | 0.0043 | 0.035 | 0.018 | 0.036 | 0.019 | 0.039 | 2.052 |
| Coprococcus_2 | 0.0043 | 0.035 | 0.0097 | 0.021 | 0.0097 | 0.02 | 2.12 |
| Anaerostipes | 0.0043 | 0.035 | 0.02 | 0.036 | 0.022 | 0.037 | 1.806 |
| Agathobacter | 0.0043 | 0.035 | 0.03 | 0.043 | 0.031 | 0.041 | 1.419 |
| Christensenellaceae_R7_group | 0.0043 | 0.035 | 0.03 | 0.017 | 0.029 | 0.017 | -1.775 |
| Leuconostoc | 0.0043 | 0.035 | 0.0009 | 0.0021 | 0.00095 | 0.0022 | 2.396 |
| Lactobacillus | 0.0043 | 0.035 | 0.00072 | 0.063 | 0.00066 | 0.062 | 86.7 |
| Parabacteroides | 0.0043 | 0.035 | 0.022 | 0.00074 | 0.023 | 0.00052 | -29.24 |
| Arthrobacter | 0.0046 | 0.037 | 0 | 0.000015 | 0 | 0.000015 | 15000 |
| Corynebacterium_1 | 0.0054 | 0.038 | 0 | 0.00011 | 0 | 0.00012 | 113300 |
| Bacillus | 0.0055 | 0.038 | 0 | 0.000098 | 0 | 0.0001 | 98330 |
| Prevotella_7 | 0.0066 | 0.038 | 0.000002 | 0.00044 | 0 | 0.00048 | 222.4 |
| Clostridium_sensu_stricto_13 | 0.0067 | 0.038 | 0.0098 | 0.000005 | 0.0012 | 0 | -1962 |
| Rikenellaceae_RC9_gut_group | 0.0074 | 0.038 | 0.001 | 0.00016 | 0.00098 | 0.00016 | -6.303 |
| Haemophilus | 0.0077 | 0.038 | 0.00025 | 0.00004 | 0.0002 | 0.00005 | -6.15 |
| Anaerococcus | 0.0078 | 0.038 | 0.001 | 0.000032 | 0.00087 | 0.000035 | -32.97 |
| Prevotella_9 | 0.0078 | 0.038 | 0.000024 | 0.00084 | 0.00002 | 0.00056 | 35 |
| Faecalitalea | 0.008 | 0.038 | 0.00034 | 0.000057 | 0.00035 | 0.00006 | -6.035 |
| Ruminococcaceae_UBA1819 | 0.008 | 0.038 | 0.00096 | 0.00033 | 0.001 | 0.00036 | -2.894 |
| Ruminiclostridium_1 | 0.008 | 0.038 | 0.0013 | 0.0003 | 0.0012 | 0.00027 | -4.544 |
| Lachnospiraceae_NC2004_group | 0.008 | 0.038 | 0.0013 | 0.0022 | 0.0013 | 0.0021 | 1.691 |
| Acetitomaculum | 0.008 | 0.038 | 0.000046 | 0.00018 | 0.00004 | 0.0002 | 3.804 |
| Peptoniphilus | 0.008 | 0.038 | 0.0049 | 0.000075 | 0.0027 | 0.000015 | -65.36 |
| Clostridiales_vadinBB60_group_uncultured_bacterium | 0.008 | 0.038 | 0.0002 | 0.000032 | 0.00019 | 0.00003 | -6.442 |
| Barnesiella | 0.008 | 0.038 | 0.0032 | 0.0008 | 0.0025 | 0.00077 | -4.024 |
| Bacteroides | 0.008 | 0.038 | 0.096 | 0.016 | 0.1 | 0.016 | -6.133 |
| Bilophila | 0.0087 | 0.038 | 0.0029 | 0.0017 | 0.0026 | 0.0017 | -1.663 |
| Turicibacter | 0.0087 | 0.038 | 0.0019 | 0.0032 | 0.0021 | 0.0033 | 1.738 |
| Ruminococcaceae_UCG005 | 0.0087 | 0.038 | 0.0075 | 0.0041 | 0.0082 | 0.0042 | -1.819 |
| Eubacterium_ventriosum_group | 0.0087 | 0.038 | 0.0035 | 0.005 | 0.0034 | 0.0051 | 1.427 |
| Christensenellaceae_uncultured | 0.0087 | 0.038 | 0.00079 | 0.00051 | 0.00082 | 0.0005 | -1.562 |
| Eggerthellaceae_Slackia | 0.0087 | 0.038 | 0.0011 | 0.0019 | 0.001 | 0.002 | 1.756 |
| Collinsella | 0.0087 | 0.038 | 0.012 | 0.022 | 0.013 | 0.021 | 1.827 |
| Fournierella | 0.01 | 0.043 | 0.000098 | 0.00002 | 0.0001 | 0.00002 | -4.9 |

Supplementary table 8. Table showing Wilcoxon rank p values, BH corrected p values, means, medians and fold changes (means) of significant phyla of 4. Enzyme treated fermented high WEAX yield treated vessels and 0.negative control treated vessels after 24 hours of fermentation.

| **Taxa.__OTU___** | **P (rank test)** | **BH corrected** | **0. Negative Control mean** | **4. Enzyme treated fermented high WEAX yield mean** | **0. Negative Control median** | **4. Enzyme treated fermented high WEAX yield median** | **Fold Change** |
| --- | --- | --- | --- | --- | --- | --- | --- |
| Bacteroidetes | 0.0043 | 0.043 | 0.14 | 0.026 | 0.14 | 0.019 | -5.263 |

Supplementary table 9. Table showing Wilcoxon rank p values, BH corrected p values, means, medians and fold changes (means) of significant phyla of 5. Enzyme treated fermented high yield grinded treated vessels and 0.negative control treated vessels after 24 hours of fermentation.

| **Taxa.__OTU___** | **P (rank test)** | **BH corrected** | **0. Negative Control mean** | **5. Enzyme treated fermented high yield grinded mean** | **0. Negative Control median** | **5. Enzyme treated fermented high yield grinded median** | **Fold Change** |
| --- | --- | --- | --- | --- | --- | --- | --- |
| Proteobacteria | 0.0043 | 0.022 | 0.04 | 0.35 | 0.016 | 0.34 | 8.886 |
| Bacteroidetes | 0.0043 | 0.022 | 0.14 | 0.011 | 0.14 | 0.0093 | -12.17 |
| Verrucomicrobia | 0.017 | 0.042 | 0.0089 | 0.0029 | 0.011 | 0.0026 | -3.036 |
| Firmicutes | 0.017 | 0.042 | 0.75 | 0.59 | 0.76 | 0.61 | -1.271 |

Supplementary table 10. Table showing Wilcoxon rank p values, BH corrected p values, means, medians and fold changes (means) of significant genera of 5. Enzyme treated fermented high yield grinded treated vessels and 0.negative control treated vessels after 24 hours of fermentation.

| **Taxa.__OTU___** | **P (rank test)** | **BH corrected** | **0. Negative Control mean** | **5. Enzyme treated fermented high yield grinded mean** | **0. Negative Control median** | **5. Enzyme treated fermented high yield grinded median** | **Fold Change** |
| --- | --- | --- | --- | --- | --- | --- | --- |
| Escherichia_Shigella | 0.0043 | 0.028 | 0.033 | 0.33 | 0.0064 | 0.33 | 9.902 |
| Dialister | 0.0043 | 0.028 | 0.0078 | 0.022 | 0.0087 | 0.021 | 2.816 |
| Phascolarctobacterium | 0.0043 | 0.028 | 0.014 | 0.065 | 0.013 | 0.062 | 4.59 |
| Holdemanella | 0.0043 | 0.028 | 0.0099 | 0.0046 | 0.011 | 0.0047 | -2.128 |
| Coprobacillus | 0.0043 | 0.028 | 0.00043 | 0.000065 | 0.00039 | 0.000065 | -6.554 |
| Ruminococcaceae_uncultured | 0.0043 | 0.028 | 0.0091 | 0.0025 | 0.01 | 0.0023 | -3.622 |
| Ruminococcaceae_UBA1819 | 0.0043 | 0.028 | 0.00096 | 0.00017 | 0.001 | 0.00017 | -5.592 |
| Ruminococcus_2 | 0.0043 | 0.028 | 0.034 | 0.0046 | 0.032 | 0.0046 | -7.316 |
| Ruminococcus_1 | 0.0043 | 0.028 | 0.0035 | 0.0019 | 0.0035 | 0.0019 | -1.832 |
| Ruminococcaceae_UCG010 | 0.0043 | 0.028 | 0.002 | 0.00092 | 0.0023 | 0.00092 | -2.179 |
| Ruminococcaceae_UCG005 | 0.0043 | 0.028 | 0.0075 | 0.0014 | 0.0082 | 0.0011 | -5.344 |
| Ruminococcaceae_UCG002 | 0.0043 | 0.028 | 0.029 | 0.0093 | 0.029 | 0.008 | -3.126 |
| Ruminiclostridium_5 | 0.0043 | 0.028 | 0.0034 | 0.0011 | 0.0036 | 0.0011 | -3.122 |
| Ruminococcaceae_GCA900066225 | 0.0043 | 0.028 | 0.00057 | 0.00025 | 0.00054 | 0.00026 | -2.249 |
| Sellimonas | 0.0043 | 0.028 | 0.00028 | 0.000085 | 0.00028 | 0.000075 | -3.341 |
| Lachnospira | 0.0043 | 0.028 | 0.0094 | 0.0025 | 0.0099 | 0.0028 | -3.685 |
| Eubacterium_eligens_group | 0.0043 | 0.028 | 0.0095 | 0.0026 | 0.0095 | 0.0027 | -3.589 |
| Blautia | 0.0043 | 0.028 | 0.063 | 0.029 | 0.066 | 0.028 | -2.206 |
| Clostridiales_Family_XIII_UCG001 | 0.0043 | 0.028 | 0.00042 | 0.00021 | 0.00039 | 0.00021 | -2.01 |
| Defluviitaleaceae_UCG011 | 0.0043 | 0.028 | 0.00059 | 0.00019 | 0.00059 | 0.00018 | -3.139 |
| Christensenellaceae_uncultured | 0.0043 | 0.028 | 0.00079 | 0.00025 | 0.00082 | 0.00022 | -3.155 |
| Christensenellaceae_R7_group | 0.0043 | 0.028 | 0.03 | 0.0087 | 0.029 | 0.0077 | -3.394 |
| Lactobacillus | 0.0043 | 0.028 | 0.00072 | 0.066 | 0.00066 | 0.07 | 91.23 |
| Carnobacterium | 0.0043 | 0.028 | 0.00032 | 0.014 | 0.00029 | 0.014 | 45.07 |
| Parabacteroides | 0.0043 | 0.028 | 0.022 | 0.00035 | 0.023 | 0.00023 | -62.13 |
| Alistipes | 0.0043 | 0.028 | 0.011 | 0.0015 | 0.012 | 0.0015 | -7.712 |
| Barnesiella | 0.0043 | 0.028 | 0.0032 | 0.00012 | 0.0025 | 0.00012 | -27.48 |
| Clavibacter | 0.0043 | 0.028 | 0 | 0.000013 | 0 | 0.00001 | 13330 |
| Corynebacterium_1 | 0.0054 | 0.028 | 0 | 0.000083 | 0 | 0.000085 | 83330 |
| Barnesiellaceae_uncultured | 0.0055 | 0.028 | 0.000082 | 0.000012 | 0.00008 | 0.00001 | -7.028 |
| Oribacterium | 0.0066 | 0.028 | 0.00011 | 0.00005 | 0.00012 | 0.00006 | -2.24 |
| Peptoniphilus | 0.0066 | 0.028 | 0.0049 | 6.7E-06 | 0.0027 | 0.00001 | -735.2 |
| Prevotella_7 | 0.0066 | 0.028 | 0.000002 | 0.00014 | 0 | 0.00011 | 70.8 |
| Haemophilus | 0.0067 | 0.028 | 0.00025 | 0.00001 | 0.0002 | 0 | -24.6 |
| Desulfovibrionaceae_uncultured | 0.0067 | 0.028 | 0.00009 | 0.000012 | 0.00009 | 0.00001 | -7.714 |
| Faecalitalea | 0.0067 | 0.028 | 0.00034 | 0.000022 | 0.00035 | 0.00002 | -15.78 |
| Finegoldia | 0.0067 | 0.028 | 0.000078 | 0.000005 | 0.00008 | 0 | -15.6 |
| Fournierella | 0.0069 | 0.028 | 0.000098 | 0.000005 | 0.0001 | 0.000005 | -19.6 |
| Butyrivibrio | 0.0073 | 0.028 | 0.00041 | 0.000012 | 0.00046 | 0.00001 | -35.31 |
| Clostridium_innocuum_group | 0.0074 | 0.028 | 0.00016 | 0.000025 | 0.00014 | 0.00003 | -6.24 |
| Ruminococcaceae_UCG007 | 0.0074 | 0.028 | 0.00013 | 0.000017 | 0.00018 | 0.00002 | -8.04 |
| Clostridiales_vadinBB60_group_uncultured_bacterium | 0.0074 | 0.028 | 0.0002 | 0.000012 | 0.00019 | 0.00001 | -17.48 |
| Clostridium_sensu_stricto_13 | 0.0074 | 0.028 | 0.0098 | 0.00001 | 0.0012 | 0.000005 | -981.3 |
| Enterobacteriaceae_Other | 0.0075 | 0.028 | 0.000006 | 0.0051 | 0 | 0.0036 | 852.6 |
| Candidatus_Soleaferrea | 0.0075 | 0.028 | 0.0011 | 0.000045 | 0.0012 | 0.00005 | -25.2 |
| Lachnospiraceae_UCG003 | 0.0075 | 0.028 | 0.0021 | 0.000025 | 0.002 | 0.00002 | -83.6 |
| Ruminococcaceae_DTU089 | 0.0077 | 0.028 | 0.00018 | 0.000055 | 0.00019 | 0.00005 | -3.345 |
| Peptococcus | 0.0077 | 0.028 | 0.00038 | 0.00003 | 0.00025 | 0.00003 | -12.53 |
| Merdibacter | 0.0078 | 0.028 | 0.00037 | 0.00007 | 0.00048 | 0.000075 | -5.314 |
| Ruminiclostridium_1 | 0.0078 | 0.028 | 0.0013 | 0.00005 | 0.0012 | 0.00004 | -26.96 |
| Lachnospiraceae_Ambiguous_taxa | 0.0078 | 0.028 | 0.00045 | 0.00019 | 0.00041 | 0.00018 | -2.368 |
| Anaerococcus | 0.0078 | 0.028 | 0.001 | 0.000015 | 0.00087 | 0.00001 | -69.6 |
| Klebsiella | 0.008 | 0.028 | 0.00002 | 0.01 | 0.00001 | 0.0038 | 500.7 |
| Holdemania | 0.008 | 0.028 | 0.00024 | 0.00005 | 0.00025 | 0.00005 | -4.76 |
| Ruminococcaceae_UCG003 | 0.008 | 0.028 | 0.0029 | 0.00026 | 0.003 | 0.00017 | -11.09 |
| Ruminiclostridium_6 | 0.008 | 0.028 | 0.0014 | 0.00014 | 0.0015 | 0.00015 | -10.32 |
| Peptococcaceae_uncultured | 0.008 | 0.028 | 0.00032 | 0.00013 | 0.00029 | 0.00012 | -2.558 |
| Tyzzerella_3 | 0.008 | 0.028 | 0.002 | 0.00016 | 0.0021 | 0.00014 | -12 |
| Lachnospiraceae_ND3007_group | 0.008 | 0.028 | 0.0083 | 0.00085 | 0.0089 | 0.00087 | -9.819 |
| Lachnospiraceae_FCS020_group | 0.008 | 0.028 | 0.0019 | 0.00045 | 0.0019 | 0.0004 | -4.231 |
| Howardella | 0.008 | 0.028 | 0.0005 | 0.00021 | 0.00051 | 0.00021 | -2.362 |
| Agathobacter | 0.008 | 0.028 | 0.03 | 0.017 | 0.031 | 0.017 | -1.728 |
| Rikenellaceae_RC9_gut_group | 0.008 | 0.028 | 0.001 | 0.000048 | 0.00098 | 0.000045 | -20.65 |
| Bacteroides | 0.008 | 0.028 | 0.096 | 0.0071 | 0.1 | 0.0056 | -13.63 |
| Coriobacteriales_Incertae_Sedis_uncultured | 0.008 | 0.028 | 0.00095 | 0.00049 | 0.001 | 0.00047 | -1.924 |
| Coriobacteriaceae_UCG003 | 0.008 | 0.028 | 0.00014 | 0.00007 | 0.00014 | 0.000075 | -2.029 |
| Marvinbryantia | 0.0087 | 0.03 | 0.0013 | 0.00073 | 0.0014 | 0.00068 | -1.855 |
| Eggerthella | 0.0087 | 0.03 | 0.00049 | 0.00011 | 0.00033 | 0.000095 | -4.436 |
| Peptostreptococcus | 0.009 | 0.03 | 0.000026 | 1.7E-06 | 0.00002 | 0 | -15.59 |
| Sutterella | 0.01 | 0.033 | 0.000048 | 0.002 | 0.00004 | 0.00096 | 41.67 |
| Veillonella | 0.01 | 0.033 | 0.00027 | 0.0011 | 0.00026 | 0.00062 | 4.317 |
| Odoribacter | 0.012 | 0.038 | 0.00016 | 0.000042 | 0.00017 | 0.00004 | -3.792 |
| Clostridia_DTU014_uncultured_bacterium | 0.013 | 0.039 | 0.00014 | 0.000012 | 0.00018 | 0.00001 | -12.17 |
| Ruminococcaceae_Other | 0.013 | 0.039 | 0.00051 | 0.00016 | 0.00058 | 0.00014 | -3.147 |
| Ruminiclostridium_9 | 0.013 | 0.039 | 0.001 | 0.00042 | 0.001 | 0.00038 | -2.52 |
| Terrisporobacter | 0.013 | 0.039 | 0.00098 | 0.0002 | 0.00037 | 0.00018 | -5.005 |
| Muribaculaceae_uncultured_bacterium | 0.013 | 0.039 | 0.0036 | 0.00058 | 0.0037 | 0.00028 | -6.148 |
| Rhodospirillales_uncultured_bacterium | 0.015 | 0.042 | 0.000044 | 0 | 0.00005 | 0 | -44000 |
| Lachnospiraceae_GCA900066755 | 0.015 | 0.042 | 0.000016 | 0 | 0.00001 | 0 | -16000 |
| Helcococcus | 0.015 | 0.042 | 0.000046 | 0 | 0.00006 | 0 | -46000 |
| Gallicola | 0.015 | 0.042 | 0.000038 | 0 | 0.00004 | 0 | -38000 |
| Ezakiella | 0.015 | 0.042 | 0.00011 | 0.000022 | 0.0001 | 0.00001 | -4.892 |
| Papillibacter | 0.016 | 0.045 | 0.000068 | 0.000013 | 0.00008 | 0.00001 | -5.1 |
| Akkermansia | 0.017 | 0.045 | 0.0089 | 0.0029 | 0.011 | 0.0026 | -3.036 |
| Ruminococcaceae_UCG014 | 0.017 | 0.045 | 0.04 | 0.025 | 0.044 | 0.023 | -1.598 |
| Ruminiclostridium | 0.017 | 0.045 | 0.00017 | 0.000058 | 0.00014 | 0.000045 | -2.88 |
| Eubacterium_ventriosum_group | 0.017 | 0.045 | 0.0035 | 0.0025 | 0.0034 | 0.0026 | -1.374 |
| Clostridiales_Family_XIII_AD3011_group | 0.017 | 0.045 | 0.0017 | 0.0012 | 0.0016 | 0.0012 | -1.443 |

Supplementary table 11. Table showing Wilcoxon rank p values, BH corrected p values, means, medians and fold changes (means) of significant phyla of 6. Insoluble fibre extract treated vessels and 0.negative control treated vessels after 24 hours of fermentation.

| **Taxa.__OTU___** | **P (rank test)** | **BH corrected** | **0. Negative Control mean** | **6. Insoluble fibre extract mean** | **0. Negative Control median** | **6. Insoluble fibre extract median** | **Fold Change** |
| --- | --- | --- | --- | --- | --- | --- | --- |
| Bacteroidetes | 0.0043 | 0.043 | 0.14 | 0.0052 | 0.14 | 0.0037 | -26.53 |

Supplementary table 12. Table showing Wilcoxon rank p values, BH corrected p values, means, medians and fold changes (means) of significant genera of 6. Insoluble fibre extract treated vessels and 0.negative control treated vessels after 24 hours of fermentation.

| **Taxa.__OTU___** | **P (rank test)** | **BH corrected** | **0. Negative Control mean** | **6. Insoluble fibre extract mean** | **0. Negative Control median** | **6. Insoluble fibre extract median** | **Fold Change** |
| --- | --- | --- | --- | --- | --- | --- | --- |
| Bilophila | 0.0043 | 0.036 | 0.0029 | 0.00071 | 0.0026 | 0.0007 | -4.107 |
| Veillonella | 0.0043 | 0.036 | 0.00027 | 0.00069 | 0.00026 | 0.00068 | 2.594 |
| Dialister | 0.0043 | 0.036 | 0.0078 | 0.023 | 0.0087 | 0.023 | 2.994 |
| Phascolarctobacterium | 0.0043 | 0.036 | 0.014 | 0.065 | 0.013 | 0.073 | 4.544 |
| Faecalitalea | 0.0043 | 0.036 | 0.00034 | 0.000043 | 0.00035 | 0.000035 | -7.892 |
| Coprobacillus | 0.0043 | 0.036 | 0.00043 | 0.00019 | 0.00039 | 0.0002 | -2.242 |
| Ruminococcaceae_uncultured | 0.0043 | 0.036 | 0.0091 | 0.0047 | 0.01 | 0.0049 | -1.919 |
| Ruminococcaceae_UBA1819 | 0.0043 | 0.036 | 0.00096 | 0.00027 | 0.001 | 0.00027 | -3.578 |
| Ruminococcus_2 | 0.0043 | 0.036 | 0.034 | 0.019 | 0.032 | 0.017 | -1.809 |
| Ruminococcaceae_UCG005 | 0.0043 | 0.036 | 0.0075 | 0.0021 | 0.0082 | 0.002 | -3.513 |
| Ruminococcaceae_UCG003 | 0.0043 | 0.036 | 0.0029 | 0.0008 | 0.003 | 0.00078 | -3.642 |
| Ruminococcaceae_UCG002 | 0.0043 | 0.036 | 0.029 | 0.014 | 0.029 | 0.014 | -2.126 |
| Ruminococcaceae_GCA900066225 | 0.0043 | 0.036 | 0.00057 | 0.00029 | 0.00054 | 0.00027 | -1.963 |
| Lachnospiraceae_UCG003 | 0.0043 | 0.036 | 0.0021 | 0.00012 | 0.002 | 0.00011 | -17.18 |
| Lachnospiraceae_ND3007_group | 0.0043 | 0.036 | 0.0083 | 0.0021 | 0.0089 | 0.0023 | -3.976 |
| Lachnospiraceae_FCS020_group | 0.0043 | 0.036 | 0.0019 | 0.00063 | 0.0019 | 0.00061 | -3.016 |
| Coprococcus_2 | 0.0043 | 0.036 | 0.0097 | 0.018 | 0.0097 | 0.018 | 1.902 |
| Christensenellaceae_R7_group | 0.0043 | 0.036 | 0.03 | 0.012 | 0.029 | 0.012 | -2.394 |
| Carnobacterium | 0.0043 | 0.036 | 0.00032 | 0.012 | 0.00029 | 0.011 | 37.88 |
| Alistipes | 0.0043 | 0.036 | 0.011 | 0.00098 | 0.012 | 0.00084 | -11.44 |
| Globicatella | 0.0053 | 0.036 | 0 | 0.00003 | 0 | 0.000025 | 30000 |
| Exiguobacterium | 0.0054 | 0.036 | 0 | 0.000032 | 0 | 0.000035 | 31670 |
| Glutamicibacter | 0.0054 | 0.036 | 0 | 0.000062 | 0 | 0.00006 | 61670 |
| Corynebacterium_1 | 0.0054 | 0.036 | 0 | 0.00014 | 0 | 0.00011 | 138300 |
| Haemophilus | 0.0066 | 0.036 | 0.00025 | 3.3E-06 | 0.0002 | 0 | -73.78 |
| Clostridium_sensu_stricto_13 | 0.0067 | 0.036 | 0.0098 | 6.7E-06 | 0.0012 | 0 | -1472 |
| Ezakiella | 0.007 | 0.036 | 0.00011 | 0.00001 | 0.0001 | 0.00001 | -10.6 |
| Barnesiellaceae_uncultured | 0.007 | 0.036 | 0.000082 | 0.000005 | 0.00008 | 0.000005 | -16.4 |
| Fournierella | 0.0073 | 0.036 | 0.000098 | 8.3E-06 | 0.0001 | 0.000005 | -11.76 |
| Odoribacter | 0.0073 | 0.036 | 0.00016 | 0.000023 | 0.00017 | 0.00001 | -6.771 |
| Coprobacter | 0.0074 | 0.036 | 0.000096 | 0.00001 | 0.00008 | 0.00001 | -9.599 |
| Enterobacteriaceae_Other | 0.0075 | 0.036 | 0.000006 | 0.0036 | 0 | 0.0029 | 593.5 |
| Clostridiales_vadinBB60_group_uncultured_bacterium | 0.0075 | 0.036 | 0.0002 | 0.000028 | 0.00019 | 0.000035 | -7.2 |
| Parabacteroides | 0.0075 | 0.036 | 0.022 | 0.00019 | 0.023 | 0.000045 | -115.4 |
| Butyrivibrio | 0.0077 | 0.036 | 0.00041 | 0.000055 | 0.00046 | 0.000055 | -7.491 |
| Holdemania | 0.0078 | 0.036 | 0.00024 | 0.000063 | 0.00025 | 0.000065 | -3.758 |
| Ruminococcaceae_DTU089 | 0.0078 | 0.036 | 0.00018 | 0.000067 | 0.00019 | 0.000075 | -2.76 |
| Lachnospiraceae_Ambiguous_taxa | 0.0078 | 0.036 | 0.00045 | 0.00021 | 0.00041 | 0.0002 | -2.158 |
| Peptoniphilus | 0.0078 | 0.036 | 0.0049 | 0.000067 | 0.0027 | 0.000045 | -73.53 |
| Butyricimonas | 0.0078 | 0.036 | 0.00048 | 0.000018 | 0.00029 | 0.000015 | -25.96 |
| Coriobacteriaceae_UCG003 | 0.0078 | 0.036 | 0.00014 | 0.00008 | 0.00014 | 0.000075 | -1.775 |
| Klebsiella | 0.008 | 0.036 | 0.00002 | 0.0074 | 0.00001 | 0.0068 | 369.3 |
| Ruminiclostridium_1 | 0.008 | 0.036 | 0.0013 | 0.00022 | 0.0012 | 0.00022 | -6.081 |
| Candidatus_Soleaferrea | 0.008 | 0.036 | 0.0011 | 0.000087 | 0.0012 | 0.00009 | -13.08 |
| Peptococcaceae_uncultured | 0.008 | 0.036 | 0.00032 | 0.00015 | 0.00029 | 0.00014 | -2.184 |
| Tyzzerella_3 | 0.008 | 0.036 | 0.002 | 0.00036 | 0.0021 | 0.0003 | -5.394 |
| Christensenellaceae_uncultured | 0.008 | 0.036 | 0.00079 | 0.00038 | 0.00082 | 0.00036 | -2.099 |
| Rikenellaceae_RC9_gut_group | 0.008 | 0.036 | 0.001 | 0.000038 | 0.00098 | 0.00004 | -26.03 |
| Muribaculaceae_uncultured_bacterium | 0.008 | 0.036 | 0.0036 | 0.00044 | 0.0037 | 0.00031 | -8.074 |
| Barnesiella | 0.008 | 0.036 | 0.0032 | 0.00028 | 0.0025 | 0.00025 | -11.45 |
| Bacteroides | 0.008 | 0.036 | 0.096 | 0.0031 | 0.1 | 0.0021 | -31.13 |
| Ruminococcus_1 | 0.0087 | 0.036 | 0.0035 | 0.0056 | 0.0035 | 0.0057 | 1.602 |
| Ruminococcaceae_UCG010 | 0.0087 | 0.036 | 0.002 | 0.0011 | 0.0023 | 0.0011 | -1.768 |
| Ruminiclostridium_5 | 0.0087 | 0.036 | 0.0034 | 0.0019 | 0.0036 | 0.0018 | -1.817 |
| Faecalibacterium | 0.0087 | 0.036 | 0.037 | 0.024 | 0.039 | 0.025 | -1.514 |
| Collinsella | 0.0087 | 0.036 | 0.012 | 0.02 | 0.013 | 0.02 | 1.657 |
| Clostridia_DTU014_uncultured_bacterium | 0.0096 | 0.038 | 0.00014 | 8.3E-06 | 0.00018 | 0.000005 | -17.04 |
| Terrisporobacter | 0.0097 | 0.038 | 0.00098 | 0.0002 | 0.00037 | 0.00021 | -4.84 |
| Clostridium_innocuum_group | 0.01 | 0.038 | 0.00016 | 0.000063 | 0.00014 | 0.000065 | -2.463 |
| Ruminiclostridium | 0.01 | 0.038 | 0.00017 | 0.000075 | 0.00014 | 0.000075 | -2.24 |
| Enorma | 0.011 | 0.041 | 0 | 8.3E-06 | 0 | 0.00001 | 8334 |
| xalobacter | 0.013 | 0.047 | 0.00019 | 0.000052 | 0.00012 | 0.000045 | -3.639 |
| Peptococcus | 0.013 | 0.047 | 0.00038 | 0.000047 | 0.00025 | 0.000045 | -8.057 |

Supplementary table 13. Table showing Wilcoxon rank p values, BH corrected p values, means, medians and fold changes (means) of significant phyla of 7. Soluble fibre extract vessels and 0.negative control treated vessels after 24 hours of fermentation.

| **Taxa.__OTU___** | **P (rank test)** | **BH corrected** | **0. Negative Control mean** | **7. Soluble fibre extract mean** | **0. Negative Control median** | **7. Soluble fibre extract median** | **Fold Change** |
| --- | --- | --- | --- | --- | --- | --- | --- |
| Bacteroidetes | 0.0043 | 0.019 | 0.14 | 0.0024 | 0.14 | 0.002 | -57.39 |
| Actinobacteria | 0.0043 | 0.019 | 0.059 | 0.19 | 0.062 | 0.15 | 3.239 |
| Tenericutes | 0.008 | 0.021 | 0.00088 | 0.00018 | 0.001 | 0.00013 | -4.878 |
| Patescibacteria | 0.0094 | 0.021 | 0.000016 | 0.000062 | 0.00001 | 0.00005 | 3.854 |
| Euryarchaeota | 0.014 | 0.025 | 0.00001 | 0 | 0.00001 | 0 | -10000 |
| Verrucomicrobia | 0.017 | 0.026 | 0.0089 | 0.0031 | 0.011 | 0.003 | -2.919 |

Supplementary table 14. Table showing Wilcoxon rank p values, BH corrected p values, means, medians and fold changes (means) of significant genera of 7. Soluble fibre extract vessels and 0.negative control treated vessels after 24 hours of fermentation.

| **Taxa.__OTU___** | **P (rank test)** | **BH corrected** | **0. Negative Control mean** | **7. Soluble fibre extract mean** | **0. Negative Control median** | **7. Soluble fibre extract median** | **Fold Change** |
| --- | --- | --- | --- | --- | --- | --- | --- |
| Flavonifractor | 0.0035 | 0.017 | 0.000092 | 0 | 0.00004 | 0 | -92000 |
| Lachnospiraceae_UCG008 | 0.0035 | 0.017 | 0.000044 | 0 | 0.00005 | 0 | -44000 |
| Ruminococcaceae_UCG011 | 0.0038 | 0.017 | 0.000058 | 0 | 0.00005 | 0 | -58000 |
| Ruminococcaceae_UCG007 | 0.0038 | 0.017 | 0.00013 | 0 | 0.00018 | 0 | -134000 |
| Harryflintia | 0.0038 | 0.017 | 0.000048 | 0 | 0.00005 | 0 | -48000 |
| Fournierella | 0.0038 | 0.017 | 0.000098 | 0 | 0.0001 | 0 | -98000 |
| Butyrivibrio | 0.0038 | 0.017 | 0.00041 | 0 | 0.00046 | 0 | -412000 |
| Negativibacillus | 0.0039 | 0.017 | 0.000046 | 0 | 0.00003 | 0 | -46000 |
| Intestinimonas | 0.0039 | 0.017 | 0.000044 | 0 | 0.00003 | 0 | -44000 |
| Ruminococcaceae_GCA900066225 | 0.0039 | 0.017 | 0.00057 | 0 | 0.00054 | 0 | -566000 |
| Ruminococcaceae_CAG352 | 0.0039 | 0.017 | 0.00017 | 0 | 0.00022 | 0 | -174000 |
| Acetanaerobacterium | 0.0039 | 0.017 | 0.00013 | 0 | 0.00007 | 0 | -126000 |
| Natranaerovirga | 0.0039 | 0.017 | 0.000068 | 0 | 0.00007 | 0 | -68000 |
| Ezakiella | 0.0039 | 0.017 | 0.00011 | 0 | 0.0001 | 0 | -106000 |
| Clostridiales_vadinBB60_group_uncultured_bacterium | 0.0039 | 0.017 | 0.0002 | 0 | 0.00019 | 0 | -204000 |
| Rikenellaceae_RC9_gut_group | 0.0039 | 0.017 | 0.001 | 0 | 0.00098 | 0 | -998000 |
| Bilophila | 0.0043 | 0.017 | 0.0029 | 0.00043 | 0.0026 | 0.00028 | -6.775 |
| Dialister | 0.0043 | 0.017 | 0.0078 | 0.0012 | 0.0087 | 0.0011 | -6.41 |
| Turicibacter | 0.0043 | 0.017 | 0.0019 | 0.0053 | 0.0021 | 0.0058 | 2.864 |
| Faecalitalea | 0.0043 | 0.017 | 0.00034 | 0.000027 | 0.00035 | 0.000025 | -12.82 |
| Coprobacillus | 0.0043 | 0.017 | 0.00043 | 0.000048 | 0.00039 | 0.000045 | -8.814 |
| Ruminococcaceae_uncultured | 0.0043 | 0.017 | 0.0091 | 0.00044 | 0.01 | 0.00045 | -20.9 |
| Ruminococcus_2 | 0.0043 | 0.017 | 0.034 | 0.0033 | 0.032 | 0.0033 | -10.35 |
| Ruminococcus_1 | 0.0043 | 0.017 | 0.0035 | 0.00068 | 0.0035 | 0.00068 | -5.063 |
| Ruminococcaceae_UCG014 | 0.0043 | 0.017 | 0.04 | 0.0057 | 0.044 | 0.0044 | -7.084 |
| Ruminococcaceae_UCG005 | 0.0043 | 0.017 | 0.0075 | 0.00011 | 0.0082 | 0.00012 | -70.14 |
| Ruminococcaceae_UCG002 | 0.0043 | 0.017 | 0.029 | 0.00063 | 0.029 | 0.00061 | -46.29 |
| Ruminococcaceae_NK4A214_group | 0.0043 | 0.017 | 0.0055 | 0.00008 | 0.0056 | 0.00007 | -68.62 |
| Ruminiclostridium_9 | 0.0043 | 0.017 | 0.001 | 0.00012 | 0.001 | 0.00013 | -8.966 |
| Ruminiclostridium_5 | 0.0043 | 0.017 | 0.0034 | 0.0012 | 0.0036 | 0.0012 | -2.876 |
| Faecalibacterium | 0.0043 | 0.017 | 0.037 | 0.0015 | 0.039 | 0.0014 | -25.47 |
| Eubacterium_coprostanoligenes_group | 0.0043 | 0.017 | 0.013 | 0.00068 | 0.014 | 0.00045 | -19.34 |
| Peptostreptococcaceae_uncultured | 0.0043 | 0.017 | 0.0039 | 0.011 | 0.0038 | 0.011 | 2.737 |
| Romboutsia | 0.0043 | 0.017 | 0.006 | 0.016 | 0.0059 | 0.016 | 2.597 |
| Paeniclostridium | 0.0043 | 0.017 | 0.00022 | 0.00061 | 0.00021 | 0.00064 | 2.798 |
| Lachnospiraceae_uncultured | 0.0043 | 0.017 | 0.0029 | 0.0013 | 0.0026 | 0.0011 | -2.299 |
| Lachnospiraceae_NK4A136_group | 0.0043 | 0.017 | 0.0017 | 0.00052 | 0.0018 | 0.00041 | -3.196 |
| Eubacterium_ventriosum_group | 0.0043 | 0.017 | 0.0035 | 0.00022 | 0.0034 | 0.00021 | -15.42 |
| Eubacterium_hallii_group | 0.0043 | 0.017 | 0.018 | 0.057 | 0.019 | 0.057 | 3.233 |
| Coprococcus_2 | 0.0043 | 0.017 | 0.0097 | 0.00053 | 0.0097 | 0.00047 | -18.31 |
| Lachnospiraceae_CAG56 | 0.0043 | 0.017 | 0.0034 | 0.000087 | 0.0034 | 0.000095 | -39.67 |
| Blautia | 0.0043 | 0.017 | 0.063 | 0.033 | 0.066 | 0.034 | -1.925 |
| Anaerostipes | 0.0043 | 0.017 | 0.02 | 0.041 | 0.022 | 0.043 | 2.014 |
| Agathobacter | 0.0043 | 0.017 | 0.03 | 0.0088 | 0.031 | 0.0081 | -3.414 |
| Clostridium_sensu_stricto_1 | 0.0043 | 0.017 | 0.01 | 0.025 | 0.009 | 0.026 | 2.441 |
| Christensenellaceae_R7_group | 0.0043 | 0.017 | 0.03 | 0.0019 | 0.029 | 0.0016 | -15.26 |
| Weissella | 0.0043 | 0.017 | 0.00027 | 0.001 | 0.00027 | 0.001 | 3.71 |
| Leuconostoc | 0.0043 | 0.017 | 0.0009 | 0.0034 | 0.00095 | 0.0035 | 3.819 |
| Carnobacterium | 0.0043 | 0.017 | 0.00032 | 0.021 | 0.00029 | 0.02 | 65.15 |
| Barnesiella | 0.0043 | 0.017 | 0.0032 | 0.00004 | 0.0025 | 0.00004 | -80.15 |
| Senegalimassilia | 0.0043 | 0.017 | 0.00052 | 0.0013 | 0.00056 | 0.0014 | 2.435 |
| Enterorhabdus | 0.0043 | 0.017 | 0.0012 | 0.0031 | 0.0013 | 0.0027 | 2.573 |
| Collinsella | 0.0043 | 0.017 | 0.012 | 0.034 | 0.013 | 0.032 | 2.807 |
| Dielma | 0.0054 | 0.017 | 0.000038 | 1.7E-06 | 0.00003 | 0 | -22.79 |
| Papillibacter | 0.0054 | 0.017 | 0.000068 | 1.7E-06 | 0.00008 | 0 | -40.78 |
| Lachnospiraceae_Ambiguous_taxa | 0.0054 | 0.017 | 0.00045 | 3.3E-06 | 0.00041 | 0 | -133.8 |
| Desulfovibrionaceae_uncultured | 0.0055 | 0.017 | 0.00009 | 3.3E-06 | 0.00009 | 0 | -26.99 |
| Holdemania | 0.0055 | 0.017 | 0.00024 | 1.7E-06 | 0.00025 | 0 | -142.7 |
| Clostridium_sensu_stricto_13 | 0.0055 | 0.017 | 0.0098 | 1.7E-06 | 0.0012 | 0 | -5885 |
| Odoribacter | 0.0061 | 0.017 | 0.00016 | 3.3E-06 | 0.00017 | 0 | -47.39 |
| Ruminiclostridium_1 | 0.0065 | 0.017 | 0.0013 | 3.3E-06 | 0.0012 | 0 | -404.3 |
| Mailhella | 0.0066 | 0.017 | 0.000052 | 3.3E-06 | 0.00004 | 0 | -15.6 |
| Ruminiclostridium | 0.0066 | 0.017 | 0.00017 | 3.3E-06 | 0.00014 | 0 | -50.39 |
| Oscillibacter | 0.0066 | 0.017 | 0.00044 | 3.3E-06 | 0.0004 | 0 | -133.2 |
| Oribacterium | 0.0066 | 0.017 | 0.00011 | 0.000025 | 0.00012 | 0.00002 | -4.48 |
| Butyricimonas | 0.0066 | 0.017 | 0.00048 | 3.3E-06 | 0.00029 | 0 | -142.8 |
| Peptococcaceae_uncultured | 0.0067 | 0.017 | 0.00032 | 0.000023 | 0.00029 | 0.00002 | -13.89 |
| Muribaculaceae_uncultured_bacterium | 0.0067 | 0.017 | 0.0036 | 0.00001 | 0.0037 | 0 | -356.6 |
| Oscillospira | 0.007 | 0.017 | 0.00026 | 0.000005 | 0.00017 | 0.000005 | -51.59 |
| Merdibacter | 0.0073 | 0.017 | 0.00037 | 0.000012 | 0.00048 | 0.000015 | -31.88 |
| Erysipelotrichaceae_UCG007 | 0.0073 | 0.017 | 0.000004 | 0.000038 | 0 | 0.000035 | 9.581 |
| Ruminococcaceae_DTU089 | 0.0073 | 0.017 | 0.00018 | 8.3E-06 | 0.00019 | 0.000005 | -22.08 |
| Ruminococcus_gauvreauii_group | 0.0073 | 0.017 | 0.00015 | 0.000022 | 0.00011 | 0.00002 | -6.923 |
| Mollicutes_RF39_Other_Other | 0.0074 | 0.017 | 0.00055 | 0.00001 | 0.00066 | 0.000005 | -54.99 |
| Oxalobacter | 0.0074 | 0.017 | 0.00019 | 6.7E-06 | 0.00012 | 0.000005 | -28.2 |
| Christensenellaceae_uncultured | 0.0074 | 0.017 | 0.00079 | 0.000018 | 0.00082 | 0.00002 | -43.31 |
| Ruminococcaceae_UBA1819 | 0.0075 | 0.017 | 0.00096 | 0.000017 | 0.001 | 0.00001 | -57.6 |
| Tyzzerella_4 | 0.0075 | 0.017 | 0.0005 | 0.00021 | 0.00045 | 0.0002 | -2.439 |
| Incertae_Sedis | 0.0075 | 0.017 | 0.00052 | 0.00002 | 0.00046 | 0.000005 | -26.2 |
| Candidatus_Soleaferrea | 0.0077 | 0.017 | 0.0011 | 0.00001 | 0.0012 | 0.00001 | -113.4 |
| Ruminococcaceae_Other | 0.0078 | 0.017 | 0.00051 | 0.000013 | 0.00058 | 0.000015 | -38.55 |
| Ruminococcaceae_UCG003 | 0.0078 | 0.017 | 0.0029 | 0.000015 | 0.003 | 0.000015 | -194.7 |
| Lachnospiraceae_UC512E3 | 0.0078 | 0.017 | 0.00028 | 0.000018 | 0.00023 | 0.00001 | -15.27 |
| Lachnospiraceae_UCG003 | 0.0078 | 0.017 | 0.0021 | 0.00005 | 0.002 | 0.000055 | -41.8 |
| Lachnospiraceae_ND3007_group | 0.0078 | 0.017 | 0.0083 | 0.00004 | 0.0089 | 0.00004 | -208.2 |
| Lachnospiraceae_NC2004_group | 0.0078 | 0.017 | 0.0013 | 0.000055 | 0.0013 | 0.000055 | -23.53 |
| Clostridiales_Family_XIII_Family_XIII_UCG001 | 0.0078 | 0.017 | 0.00042 | 0.000043 | 0.00039 | 0.00003 | -9.738 |
| Defluviitaleaceae_UCG011 | 0.0078 | 0.017 | 0.00059 | 0.000032 | 0.00059 | 0.00003 | -18.5 |
| Coprobacter | 0.0078 | 0.017 | 0.000096 | 0.000013 | 0.00008 | 0.000015 | -7.2 |
| Bacteroides | 0.0078 | 0.017 | 0.096 | 0.0019 | 0.1 | 0.0011 | -51.01 |
| Gordonibacter | 0.0078 | 0.017 | 0.000058 | 0.00021 | 0.00007 | 0.00022 | 3.621 |
| Veillonella | 0.008 | 0.017 | 0.00027 | 0.000043 | 0.00026 | 0.00003 | -6.138 |
| Ruminococcaceae_UCG010 | 0.008 | 0.017 | 0.002 | 0.000053 | 0.0023 | 0.000055 | -37.8 |
| Ruminiclostridium_6 | 0.008 | 0.017 | 0.0014 | 0.000025 | 0.0015 | 0.000025 | -57.12 |
| Butyricicoccus | 0.008 | 0.017 | 0.0015 | 0.00017 | 0.0016 | 0.00011 | -8.847 |
| Roseburia | 0.008 | 0.017 | 0.0027 | 0.00021 | 0.0031 | 0.0002 | -12.54 |
| Marvinbryantia | 0.008 | 0.017 | 0.0013 | 0.0003 | 0.0014 | 0.00032 | -4.444 |
| Lachnospiraceae_FCS020_group | 0.008 | 0.017 | 0.0019 | 0.00011 | 0.0019 | 0.00009 | -17.45 |
| Lachnospira | 0.008 | 0.017 | 0.0094 | 0.0002 | 0.0099 | 0.0002 | -46.12 |
| Lachnospiraceae_GCA900066575 | 0.008 | 0.017 | 0.00021 | 0.000033 | 0.00022 | 0.000035 | -6.42 |
| Eubacterium_eligens_group | 0.008 | 0.017 | 0.0095 | 0.00016 | 0.0095 | 0.0001 | -59.44 |
| Peptoniphilus | 0.008 | 0.017 | 0.0049 | 0.000028 | 0.0027 | 0.000025 | -173 |
| Parabacteroides | 0.008 | 0.017 | 0.022 | 0.00016 | 0.023 | 0.00009 | -137.5 |
| Alistipes | 0.008 | 0.017 | 0.011 | 0.00023 | 0.012 | 0.0001 | -49.2 |
| Lactobacillus | 0.0087 | 0.018 | 0.00072 | 0.0019 | 0.00066 | 0.0018 | 2.588 |
| Eubacterium_brachy_group | 0.0094 | 0.019 | 0.00011 | 0.00002 | 0.00008 | 0.000015 | -5.5 |
| Eggerthellaceae_uncultured | 0.0097 | 0.02 | 0.000008 | 0.000047 | 0.00001 | 0.000035 | 5.833 |
| Clostridia_DTU014_uncultured_bacterium | 0.01 | 0.02 | 0.00014 | 0.000015 | 0.00018 | 0.000015 | -9.466 |
| Actinomyces | 0.01 | 0.02 | 0.00012 | 0.00039 | 0.0001 | 0.00036 | 3.305 |
| Ruminococcaceae_UCG004 | 0.013 | 0.025 | 0.00033 | 0.000098 | 0.00032 | 0.00007 | -3.376 |
| Peptococcus | 0.013 | 0.025 | 0.00038 | 0.000032 | 0.00025 | 0.000025 | -11.87 |
| Eubacterium_xylanophilum_group | 0.013 | 0.025 | 0.00034 | 0.00009 | 0.00039 | 0.00008 | -3.822 |
| Clostridiales_vadinBB60_group_Other | 0.013 | 0.025 | 0.000002 | 0.000017 | 0 | 0.000015 | 8.33 |
| Eubacterium_ruminantium_group | 0.014 | 0.027 | 0.00002 | 0 | 0.00003 | 0 | -20000 |
| Methanobrevibacter | 0.014 | 0.027 | 0.00001 | 0 | 0.00001 | 0 | -10000 |
| Mollicutes_RF39_Ambiguous_taxa | 0.015 | 0.027 | 0.000014 | 0 | 0.00001 | 0 | -14000 |
| Saccharimonadaceae_uncultured_bacterium | 0.015 | 0.027 | 0.000016 | 0.000057 | 0.00001 | 0.000045 | 3.542 |
| Ruminococcaceae_UCG009 | 0.015 | 0.027 | 0.000042 | 0 | 0.00004 | 0 | -42000 |
| Anaerotruncus | 0.015 | 0.027 | 0.000042 | 0 | 0.00003 | 0 | -42000 |
| Lachnospiraceae_GCA900066755 | 0.015 | 0.027 | 0.000016 | 0 | 0.00001 | 0 | -16000 |
| Helcococcus | 0.015 | 0.027 | 0.000046 | 0 | 0.00006 | 0 | -46000 |
| Flavobacteriaceae_uncultured | 0.015 | 0.027 | 0.000076 | 0 | 0.00008 | 0 | -76000 |
| Clostridioides | 0.016 | 0.028 | 0 | 0.000028 | 0 | 0.000015 | 28330 |
| Akkermansia | 0.017 | 0.029 | 0.0089 | 0.0031 | 0.011 | 0.003 | -2.918 |
| Desulfovibrio | 0.017 | 0.029 | 0.00066 | 0.00019 | 0.00075 | 0.00016 | -3.463 |
| Tyzzerella_3 | 0.017 | 0.029 | 0.002 | 0.0008 | 0.0021 | 0.0006 | -2.455 |
| Bifidobacterium | 0.017 | 0.029 | 0.042 | 0.15 | 0.044 | 0.11 | 3.495 |
| Haemophilus | 0.021 | 0.035 | 0.00025 | 0.000037 | 0.0002 | 0.00001 | -6.709 |
| Barnesiellaceae_uncultured | 0.021 | 0.035 | 0.000082 | 0.000023 | 0.00008 | 0.000025 | -3.514 |
| Eggerthellaceae_Other | 0.021 | 0.035 | 0.00017 | 0.0004 | 0.00014 | 0.00034 | 2.382 |
| Howardella | 0.028 | 0.046 | 0.0005 | 0.00023 | 0.00051 | 0.00026 | -2.126 |
| Clostridiales_Family_XIII_S5A14a | 0.028 | 0.046 | 0.00003 | 1.7E-06 | 0.00003 | 0 | -17.99 |
| Clostridiales_Family_XIII_Family_XIII_AD3011_group | 0.028 | 0.046 | 0.0017 | 0.0012 | 0.0016 | 0.0012 | -1.431 |
| Gallicola | 0.029 | 0.047 | 0.000038 | 1.7E-06 | 0.00004 | 0 | -22.79 |
| Fusicatenibacter | 0.03 | 0.048 | 0.015 | 0.0089 | 0.015 | 0.01 | -1.624 |
| Coriobacteriales_uncultured_uncultured_bacterium | 0.03 | 0.048 | 0.000086 | 0.00023 | 0.00008 | 0.0002 | 2.694 |
| Coriobacteriales_Incertae_Sedis_uncultured | 0.03 | 0.048 | 0.00095 | 0.002 | 0.001 | 0.0018 | 2.156 |
| Enterobacteriaceae_Other | 0.032 | 0.051 | 0.000006 | 0.00066 | 0 | 0.00015 | 110.5 |
| Lactococcus | 0.035 | 0.055 | 0.00016 | 0.00037 | 0.00017 | 0.00036 | 2.312 |
| Rhodospirillales_uncultured_uncultured_bacterium | 0.043 | 0.067 | 0.000044 | 3.3E-06 | 0.00005 | 0 | -13.2 |
| Slackia | 0.044 | 0.068 | 0.0011 | 0.0017 | 0.001 | 0.0019 | 1.632 |
| Paraprevotella | 0.054 | 0.083 | 0.00089 | 0.000027 | 0.00062 | 0.000035 | -33.45 |
| Anaerofilum | 0.066 | 0.1 | 0.000046 | 3.3E-06 | 0.00004 | 0 | -13.8 |
| Klebsiella | 0.067 | 0.1 | 0.00002 | 0.0035 | 0.00001 | 0.00026 | 177.2 |
| Sutterella | 0.067 | 0.1 | 0.000048 | 0.00012 | 0.00004 | 0.00011 | 2.535 |
| Mollicutes_RF39_uncultured_bacterium | 0.082 | 0.12 | 0.00031 | 0.00017 | 0.0003 | 0.00012 | -1.842 |
| Clostridium_innocuum_group | 0.082 | 0.12 | 0.00016 | 0.000083 | 0.00014 | 0.00004 | -1.872 |
| Anaerococcus | 0.082 | 0.12 | 0.001 | 0.00037 | 0.00087 | 0.00026 | -2.822 |
| Corynebacterium | 0.084 | 0.12 | 0.000004 | 0.000015 | 0 | 0.000015 | 3.749 |
| Saccharimonadales_uncultured_bacterium | 0.099 | 0.14 | 0 | 0.000005 | 0 | 0.000005 | 5001 |
| Peptostreptococcus | 0.11 | 0.16 | 0.000026 | 0.00001 | 0.00002 | 0.00001 | -2.6 |
| Puniceicoccaceae_uncultured | 0.13 | 0.18 | 0.000008 | 0 | 0 | 0 | -8001 |
| Izimaplasmatales_Other_Other | 0.13 | 0.18 | 0.000004 | 0 | 0 | 0 | -4001 |
| Coprococcus_1 | 0.13 | 0.18 | 0.0028 | 0.002 | 0.0028 | 0.002 | -1.409 |
| Streptococcus | 0.13 | 0.18 | 0.012 | 0.023 | 0.014 | 0.025 | 1.921 |
| Enterococcus | 0.13 | 0.18 | 0.12 | 0.26 | 0.1 | 0.29 | 2.224 |
| Alloscardovia | 0.13 | 0.18 | 0.000002 | 0.000012 | 0 | 0.00001 | 5.831 |
| Lachnospiraceae_UCG010 | 0.14 | 0.19 | 0.000006 | 0 | 0 | 0 | -6001 |
| Murdochiella | 0.14 | 0.19 | 0.00001 | 1.7E-06 | 0.00001 | 0 | -5.997 |
| Parvimonas | 0.15 | 0.21 | 0.000002 | 8.3E-06 | 0 | 0.00001 | 4.165 |
| Hydrogenoanaerobacterium | 0.16 | 0.22 | 0.000012 | 1.7E-06 | 0.00001 | 0 | -7.196 |
| Parasutterella | 0.17 | 0.23 | 0.0019 | 0.0018 | 0.0019 | 0.00008 | -1.071 |
| Coriobacteriaceae_UCG003 | 0.17 | 0.23 | 0.00014 | 0.00011 | 0.00014 | 0.0001 | -1.253 |
| Globicatella | 0.22 | 0.29 | 0 | 0.000013 | 0 | 0 | 13330 |
| Enorma | 0.22 | 0.29 | 0 | 0.000005 | 0 | 0 | 5001 |
| Scardovia | 0.22 | 0.29 | 0 | 3.3E-06 | 0 | 0 | 3334 |
| Olsenella | 0.23 | 0.3 | 0.000056 | 0.000035 | 0.00007 | 0.00003 | -1.6 |
| Holdemanella | 0.25 | 0.32 | 0.0099 | 0.0074 | 0.011 | 0.0071 | -1.34 |
| Terrisporobacter | 0.25 | 0.32 | 0.00098 | 0.00065 | 0.00037 | 0.0006 | -1.505 |
| Caproiciproducens | 0.29 | 0.37 | 0.00002 | 0.000005 | 0.00002 | 0.000005 | -3.999 |
| Acetitomaculum | 0.29 | 0.37 | 0.000046 | 0.00003 | 0.00004 | 0.00003 | -1.533 |
| Caldicoprobacter | 0.29 | 0.37 | 0.000008 | 3.3E-06 | 0.00001 | 0 | -2.4 |
| Lachnospiraceae_Other | 0.33 | 0.42 | 0.026 | 0.028 | 0.027 | 0.033 | 1.109 |
| Eggerthella | 0.33 | 0.42 | 0.00049 | 0.0007 | 0.00033 | 0.00066 | 1.428 |
| Ruminococcaceae_UCG008 | 0.35 | 0.43 | 0.00001 | 1.7E-06 | 0 | 0 | -5.997 |
| Gemella | 0.35 | 0.43 | 0.00001 | 0.000018 | 0 | 0.000015 | 1.833 |
| Atopobiaceae_uncultured | 0.35 | 0.43 | 0.000066 | 0.000083 | 0.00006 | 0.00009 | 1.263 |
| Dehalobacterium | 0.36 | 0.44 | 0.000002 | 0 | 0 | 0 | -2001 |
| Eisenbergiella | 0.36 | 0.44 | 0.000004 | 0 | 0 | 0 | -4001 |
| Clostridiales_Family_XIII_uncultured | 0.36 | 0.44 | 0.000002 | 0 | 0 | 0 | -2001 |

Supplementary table 15. Table showing Wilcoxon rank p values, BH corrected p values, means, medians and fold changes (means) of significant phyla of 8. BioActor Naxus vessels and 0.negative control treated vessels after 24 hours of fermentation.

| **Taxa.__OTU___** | **P (rank test)** | **BH corrected** | **0. Negative Control mean** | **8. BioActor Naxus mean** | **0. Negative Control median** | **8. BioActor Naxus median** | **Fold Change** |
| --- | --- | --- | --- | --- | --- | --- | --- |
| Euryarchaeota | 0.0084 | 0.022 | 0.00001 | 0 | 0.00001 | 0 | -10000 |
| Tenericutes | 0.0092 | 0.022 | 0.00088 | 0.00021 | 0.001 | 0.00017 | -4.097 |
| Verrucomicrobia | 0.01 | 0.022 | 0.0089 | 0.0024 | 0.011 | 0.0014 | -3.796 |
| Proteobacteria | 0.01 | 0.022 | 0.04 | 0.29 | 0.016 | 0.32 | 7.221 |

Supplementary table 16. Table showing Wilcoxon rank p values, BH corrected p values, means, medians and fold changes (means) of significant genera of 8. BioActor Naxus vessels and 0.negative control treated vessels after 24 hours of fermentation.

| **Taxa.__OTU___** | **P (rank test)** | **BH corrected** | **0. Negative Control mean** | **8. BioActor Naxus mean** | **0. Negative Control median** | **8. BioActor Naxus median** | **Fold Change** |
| --- | --- | --- | --- | --- | --- | --- | --- |
| Peptostreptococcus | 0.002 | 0.016 | 0.000026 | 0 | 0.00002 | 0 | -26000 |
| Sutterella | 0.0025 | 0.016 | 0.000048 | 0.0039 | 0.00004 | 0.0012 | 81.52 |
| Bilophila | 0.0025 | 0.016 | 0.0029 | 0.001 | 0.0026 | 0.0011 | -2.898 |
| Phascolarctobacterium | 0.0025 | 0.016 | 0.014 | 0.057 | 0.013 | 0.062 | 3.99 |
| Turicibacter | 0.0025 | 0.016 | 0.0019 | 0.00061 | 0.0021 | 0.00058 | -3.06 |
| Ruminococcaceae_uncultured | 0.0025 | 0.016 | 0.0091 | 0.0012 | 0.01 | 0.00092 | -7.65 |
| Subdoligranulum | 0.0025 | 0.016 | 0.047 | 0.012 | 0.048 | 0.011 | -3.875 |
| Ruminococcus_2 | 0.0025 | 0.016 | 0.034 | 0.0071 | 0.032 | 0.0057 | -4.758 |
| Ruminococcaceae_UCG010 | 0.0025 | 0.016 | 0.002 | 0.00032 | 0.0023 | 0.00013 | -6.357 |
| Ruminococcaceae_UCG005 | 0.0025 | 0.016 | 0.0075 | 0.00065 | 0.0082 | 0.00019 | -11.51 |
| Ruminococcaceae_UCG002 | 0.0025 | 0.016 | 0.029 | 0.0046 | 0.029 | 0.0046 | -6.357 |
| Ruminococcaceae_GCA900066225 | 0.0025 | 0.016 | 0.00057 | 0.00013 | 0.00054 | 0.00009 | -4.402 |
| Faecalibacterium | 0.0025 | 0.016 | 0.037 | 0.0048 | 0.039 | 0.0017 | -7.76 |
| Romboutsia | 0.0025 | 0.016 | 0.006 | 0.0022 | 0.0059 | 0.0018 | -2.751 |
| Marvinbryantia | 0.0025 | 0.016 | 0.0013 | 0.0002 | 0.0014 | 0.0002 | -6.788 |
| Lachnospiraceae_UCG003 | 0.0025 | 0.016 | 0.0021 | 0.0002 | 0.002 | 0.00006 | -10.38 |
| Lachnospiraceae_NK4A136_group | 0.0025 | 0.016 | 0.0017 | 0.00045 | 0.0018 | 0.00032 | -3.741 |
| Lachnospiraceae_ND3007_group | 0.0025 | 0.016 | 0.0083 | 0.00028 | 0.0089 | 0.00009 | -29.6 |
| Lachnospira | 0.0025 | 0.016 | 0.0094 | 0.0022 | 0.0099 | 0.0017 | -4.31 |
| Fusicatenibacter | 0.0025 | 0.016 | 0.015 | 0.0045 | 0.015 | 0.0046 | -3.197 |
| Eubacterium_eligens_group | 0.0025 | 0.016 | 0.0095 | 0.0021 | 0.0095 | 0.0014 | -4.507 |
| Anaerostipes | 0.0025 | 0.016 | 0.02 | 0.0057 | 0.022 | 0.005 | -3.525 |
| Agathobacter | 0.0025 | 0.016 | 0.03 | 0.0058 | 0.031 | 0.0034 | -5.223 |
| Clostridiales_Family_XIII_Family_XIII_UCG001 | 0.0025 | 0.016 | 0.00042 | 0.000064 | 0.00039 | 0.00006 | -6.564 |
| Clostridiales_Family_XIII_Family_XIII_AD3011_group | 0.0025 | 0.016 | 0.0017 | 0.00029 | 0.0016 | 0.00024 | -5.729 |
| Defluviitaleaceae_UCG011 | 0.0025 | 0.016 | 0.00059 | 0.0002 | 0.00059 | 0.00017 | -2.869 |
| Clostridium_sensu_stricto_1 | 0.0025 | 0.016 | 0.01 | 0.0035 | 0.009 | 0.0034 | -2.926 |
| Christensenellaceae_R7_group | 0.0025 | 0.016 | 0.03 | 0.0029 | 0.029 | 0.0017 | -10.36 |
| Streptococcus | 0.0025 | 0.016 | 0.012 | 0.0034 | 0.014 | 0.0034 | -3.53 |
| Parabacteroides | 0.0025 | 0.016 | 0.022 | 0.0035 | 0.023 | 0.0031 | -6.072 |
| Alistipes | 0.0025 | 0.016 | 0.011 | 0.0018 | 0.012 | 0.0015 | -6.129 |
| Barnesiella | 0.0025 | 0.016 | 0.0032 | 0.00047 | 0.0025 | 0.00031 | -6.821 |
| Enterorhabdus | 0.0025 | 0.016 | 0.0012 | 0.0005 | 0.0013 | 0.00056 | -2.413 |
| Ruminococcaceae_UCG007 | 0.0031 | 0.018 | 0.00013 | 2.9E-06 | 0.00018 | 0 | -46.88 |
| Ezakiella | 0.0032 | 0.018 | 0.00011 | 1.4E-06 | 0.0001 | 0 | -74.15 |
| Clostridium_sensu_stricto_13 | 0.0032 | 0.018 | 0.0098 | 4.3E-06 | 0.0012 | 0 | -2289 |
| Clostridiales_vadinBB60_group_uncultured_bacterium | 0.0049 | 0.018 | 0.0002 | 0.000016 | 0.00019 | 0 | -12.98 |
| Eggerthellaceae_Other | 0.005 | 0.018 | 0.00017 | 0.000081 | 0.00014 | 0.00009 | -2.088 |
| Ruminococcaceae_UBA1819 | 0.0051 | 0.018 | 0.00096 | 0.00026 | 0.001 | 0.0002 | -3.754 |
| Ruminococcaceae_UCG013 | 0.0051 | 0.018 | 0.0043 | 0.0018 | 0.0046 | 0.0015 | -2.454 |
| Eubacterium_coprostanoligenes_group | 0.0051 | 0.018 | 0.013 | 0.0026 | 0.014 | 0.0014 | -5.003 |
| Peptostreptococcaceae_uncultured | 0.0051 | 0.018 | 0.0039 | 0.0016 | 0.0038 | 0.0015 | -2.441 |
| Lachnospiraceae_Other | 0.0051 | 0.018 | 0.026 | 0.011 | 0.027 | 0.0082 | -2.397 |
| Lachnospiraceae_GCA900066575 | 0.0051 | 0.018 | 0.00021 | 0.000054 | 0.00022 | 0.00003 | -3.942 |
| Blautia | 0.0051 | 0.018 | 0.063 | 0.02 | 0.066 | 0.014 | -3.237 |
| Muribaculaceae_uncultured_bacterium | 0.0051 | 0.018 | 0.0036 | 0.00047 | 0.0037 | 0.0002 | -7.61 |
| Papillibacter | 0.0052 | 0.018 | 0.000068 | 2.9E-06 | 0.00008 | 0 | -23.79 |
| Faecalitalea | 0.0054 | 0.018 | 0.00034 | 0.000019 | 0.00035 | 0.00001 | -18.41 |
| Oscillospira | 0.0054 | 0.018 | 0.00026 | 0.000017 | 0.00017 | 0 | -15.05 |
| Peptococcaceae_uncultured | 0.0054 | 0.018 | 0.00032 | 0.000039 | 0.00029 | 0.00002 | -8.4 |
| Lachnospiraceae_Ambiguous_taxa | 0.0055 | 0.018 | 0.00045 | 0.000037 | 0.00041 | 0.00003 | -12.01 |
| Holdemania | 0.0056 | 0.018 | 0.00024 | 0.000031 | 0.00025 | 0.00004 | -7.573 |
| Ruminiclostridium_1 | 0.0056 | 0.018 | 0.0013 | 0.000073 | 0.0012 | 0.00002 | -18.5 |
| Tyzzerella_3 | 0.0056 | 0.018 | 0.002 | 0.00021 | 0.0021 | 0.00021 | -9.462 |
| Butyrivibrio | 0.0056 | 0.018 | 0.00041 | 0.000049 | 0.00046 | 0.00002 | -8.482 |
| Lactococcus | 0.0056 | 0.018 | 0.00016 | 0.00004 | 0.00017 | 0.00004 | -4 |
| Coprobacillus | 0.0057 | 0.018 | 0.00043 | 0.000047 | 0.00039 | 0.00003 | -9.036 |
| Ruminococcaceae_Other | 0.0057 | 0.018 | 0.00051 | 0.00012 | 0.00058 | 0.00012 | -4.283 |
| Ruminococcaceae_UCG003 | 0.0057 | 0.018 | 0.0029 | 0.000093 | 0.003 | 0.00004 | -31.45 |
| Ruminiclostridium_5 | 0.0057 | 0.018 | 0.0034 | 0.00059 | 0.0036 | 0.00053 | -5.773 |
| Candidatus_Soleaferrea | 0.0057 | 0.018 | 0.0011 | 0.0001 | 0.0012 | 0.0001 | -10.87 |
| Lachnospiraceae_FCS020_group | 0.0057 | 0.018 | 0.0019 | 0.00018 | 0.0019 | 0.00013 | -10.42 |
| Howardella | 0.0057 | 0.018 | 0.0005 | 0.00012 | 0.00051 | 0.00009 | -3.991 |
| Coprococcus_1 | 0.0057 | 0.018 | 0.0028 | 0.00052 | 0.0028 | 0.00052 | -5.283 |
| Christensenellaceae_uncultured | 0.0057 | 0.018 | 0.00079 | 0.00015 | 0.00082 | 0.00011 | -5.243 |
| Slackia | 0.0057 | 0.018 | 0.0011 | 0.00059 | 0.001 | 0.00061 | -1.812 |
| Eggerthella | 0.0057 | 0.018 | 0.00049 | 0.000093 | 0.00033 | 0.00009 | -5.255 |
| Coriobacteriales_Incertae_Sedis_uncultured | 0.0057 | 0.018 | 0.00095 | 0.00035 | 0.001 | 0.00029 | -2.736 |
| Atopobiaceae_uncultured | 0.0061 | 0.019 | 0.000066 | 0.00001 | 0.00006 | 0.00001 | -6.599 |
| Ruminiclostridium | 0.0072 | 0.022 | 0.00017 | 0.000037 | 0.00014 | 0.00002 | -4.523 |
| Mogibacterium | 0.0083 | 0.024 | 0.000052 | 8.6E-06 | 0.00004 | 0.00001 | -6.066 |
| Methanobrevibacter | 0.0084 | 0.024 | 0.00001 | 0 | 0.00001 | 0 | -10000 |
| Oribacterium | 0.0088 | 0.024 | 0.00011 | 0.000041 | 0.00012 | 0.00004 | -2.703 |
| Mollicutes_RF39_Other_Other | 0.0089 | 0.024 | 0.00055 | 0.000094 | 0.00066 | 0.00004 | -5.833 |
| Lachnospiraceae_GCA900066755 | 0.0089 | 0.024 | 0.000016 | 0 | 0.00001 | 0 | -16000 |
| Clostridiales_Family_XIII_S5A14a | 0.0089 | 0.024 | 0.00003 | 0 | 0.00003 | 0 | -30000 |
| Helcococcus | 0.0089 | 0.024 | 0.000046 | 0 | 0.00006 | 0 | -46000 |
| Clostridiales_vadinBB60_group_Ambiguous_taxa | 0.0089 | 0.024 | 0.000022 | 0 | 0.00001 | 0 | -22000 |
| Ruminococcaceae_DTU089 | 0.0091 | 0.024 | 0.00018 | 0.000064 | 0.00019 | 0.00004 | -2.862 |
| Lachnospiraceae_NC2004_group | 0.0091 | 0.024 | 0.0013 | 0.00019 | 0.0013 | 0.00006 | -6.862 |
| Ruminiclostridium_6 | 0.0092 | 0.024 | 0.0014 | 0.00027 | 0.0015 | 0.00013 | -5.317 |
| Tyzzerella_4 | 0.0092 | 0.024 | 0.0005 | 0.00016 | 0.00045 | 0.00013 | -3.122 |
| Akkermansia | 0.01 | 0.024 | 0.0089 | 0.0024 | 0.011 | 0.0014 | -3.804 |
| EscherichiaShigella | 0.01 | 0.024 | 0.033 | 0.27 | 0.0064 | 0.32 | 8.216 |
| Ruminococcaceae_UCG014 | 0.01 | 0.024 | 0.04 | 0.015 | 0.044 | 0.0092 | -2.704 |
| Ruminococcaceae_NK4A214_group | 0.01 | 0.024 | 0.0055 | 0.0012 | 0.0056 | 0.00031 | -4.658 |
| Ruminiclostridium_9 | 0.01 | 0.024 | 0.001 | 0.00028 | 0.001 | 0.00022 | -3.794 |
| Natranaerovirga | 0.01 | 0.024 | 0.000068 | 0.000014 | 0.00007 | 0 | -4.76 |
| Eubacterium_xylanophilum_group | 0.01 | 0.024 | 0.00034 | 0.000041 | 0.00039 | 0.00001 | -8.303 |
| Fournierella | 0.011 | 0.026 | 0.000098 | 0.00001 | 0.0001 | 0 | -9.799 |
| Klebsiella | 0.012 | 0.028 | 0.00002 | 0.0037 | 0.00001 | 0.00024 | 185.6 |
| Ruminococcaceae_UCG004 | 0.012 | 0.028 | 0.00033 | 0.000063 | 0.00032 | 0.00004 | -5.282 |
| Peptococcus | 0.012 | 0.028 | 0.00038 | 0.000047 | 0.00025 | 0.00006 | -7.976 |
| Sellimonas | 0.012 | 0.028 | 0.00028 | 0.000083 | 0.00028 | 0.00007 | -3.428 |
| Clostridia_DTU014_uncultured_bacterium | 0.014 | 0.032 | 0.00014 | 0.000016 | 0.00018 | 0.00001 | -9.036 |
| Coprobacter | 0.014 | 0.032 | 0.000096 | 0.000021 | 0.00008 | 0.00001 | -4.48 |
| Mollicutes_RF39_uncultured_bacterium | 0.015 | 0.032 | 0.00031 | 0.00011 | 0.0003 | 0.00013 | -2.712 |
| Oscillibacter | 0.015 | 0.032 | 0.00044 | 0.000063 | 0.0004 | 0.00004 | -7.064 |
| Roseburia | 0.015 | 0.032 | 0.0027 | 0.00056 | 0.0031 | 0.00014 | -4.791 |
| Rikenellaceae_RC9_gut_group | 0.015 | 0.032 | 0.001 | 0.00014 | 0.00098 | 0.00004 | -6.917 |
| Haemophilus | 0.016 | 0.034 | 0.00025 | 0.000046 | 0.0002 | 0 | -5.381 |
| Ruminococcus_gauvreauii_group | 0.017 | 0.036 | 0.00015 | 0.000043 | 0.00011 | 0.00004 | -3.5 |
| Allisonella | 0.018 | 0.036 | 0.000004 | 0.00089 | 0 | 0.00016 | 222.8 |
| Butyricicoccus | 0.018 | 0.036 | 0.0015 | 0.00056 | 0.0016 | 0.00025 | -2.699 |
| Paeniclostridium | 0.018 | 0.036 | 0.00022 | 0.000073 | 0.00021 | 0.00006 | -2.992 |
| Lachnospiraceae_UC512E3 | 0.018 | 0.036 | 0.00028 | 0.00012 | 0.00023 | 0.00013 | -2.306 |
| Lachnoclostridium | 0.018 | 0.036 | 0.012 | 0.0068 | 0.013 | 0.0067 | -1.742 |
| Eubacterium_hallii_group | 0.018 | 0.036 | 0.018 | 0.01 | 0.019 | 0.0092 | -1.752 |
| Lachnospiraceae_CAG56 | 0.018 | 0.036 | 0.0034 | 0.00084 | 0.0034 | 0.00045 | -4.1 |
| Ruminococcaceae_UCG009 | 0.019 | 0.037 | 0.000042 | 1.4E-06 | 0.00004 | 0 | -29.38 |
| Lachnospiraceae_UCG008 | 0.02 | 0.039 | 0.000044 | 0.00001 | 0.00005 | 0.00001 | -4.4 |
| Ruminococcaceae_CAG352 | 0.022 | 0.042 | 0.00017 | 0.000026 | 0.00022 | 0.00001 | -6.766 |
| Eubacterium_brachy_group | 0.022 | 0.042 | 0.00011 | 0.000021 | 0.00008 | 0.00001 | -5.133 |
| Anaerotruncus | 0.023 | 0.043 | 0.000042 | 2.9E-06 | 0.00003 | 0 | -14.7 |
| Leuconostoc | 0.023 | 0.043 | 0.0009 | 0.00044 | 0.00095 | 0.00039 | -2.017 |

Supplementary table 17. Table showing Wilcoxon rank p values, BH corrected p values, means, medians and fold changes (means) of significant phyla of 5. Enzyme treated fermented high yield grinded and 4. Enzyme treated fermented high WEAX yield treated vessels after 24 hours of fermentation.

| **Taxa.__OTU___** | **P (rank test)** | **BH corrected** | **4. Enzyme treated fermented high WEAX yield mean** | **5. Enzyme treated fermented high yield grinded mean** | **4. Enzyme treated fermented high WEAX yield median** | **5. Enzyme treated fermented high yield grinded median** | **Fold Change** |
| --- | --- | --- | --- | --- | --- | --- | --- |
| Firmicutes | 0.0022 | 0.022 | 0.86 | 0.59 | 0.88 | 0.61 | -1.455 |
| Proteobacteria | 0.005 | 0.025 | 0.023 | 0.35 | 0.012 | 0.34 | 15.25 |
